# Supplementary material for: Nitrogen and CO2 enrichment interact to decrease biodiversity impact on complementarity and selection effects
Source: Nat Commun. 2025 Aug 12;16:7445. doi: 10.1038/s41467-025-62691-0 (PMC12343814; doi:10.1038/s41467-025-62691-0)
Supplement: Supplementary file 1 — Supplementary Information [file 41467_2025_62691_MOESM1_ESM.pdf]

Nitrogen and CO_2 enrichment interact to decrease biodiversity impact on complementarity and selection effects

Mengjiao Huang et al.

Supplementary Figure 1. Linear mixed-effects models followed by type III ANOVA for the effects of eCO_2 and nitrogen (N) addition on the relationships between mean of community productivity and (a) complementarity effect (CE) and (b) selection effect (SE), between community stability and (c) species asynchrony (asy), (d) species stability (spp_stab) and (e) productivity (mean_prod), with rings as the random intercept. ***$P < 0.001$, **$P < 0.01$, *$P < 0.05$, .$P < 0.1$. The significant predictors are indicated by text in the figures. Each point represents a plot.

Supplementary Figure 1.

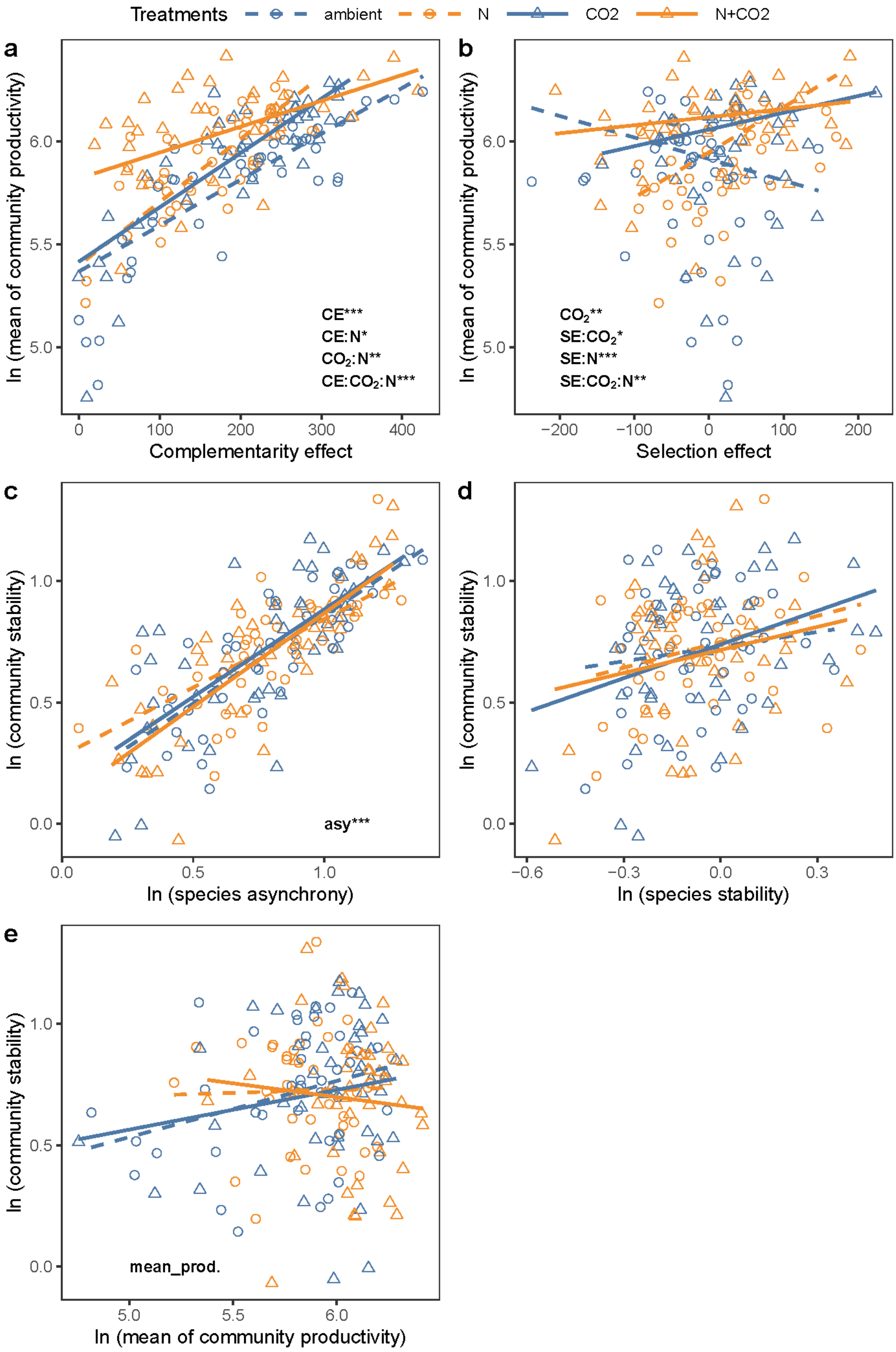

Supplementary Figure 2. Partial regression plots of the relationship between (a) species asynchrony and complementarity effect (CE), and between (b) species asynchrony and selection effect (SE) after controlling the interactive effect between species richness, N and eCO_2. Each point represents a plot.

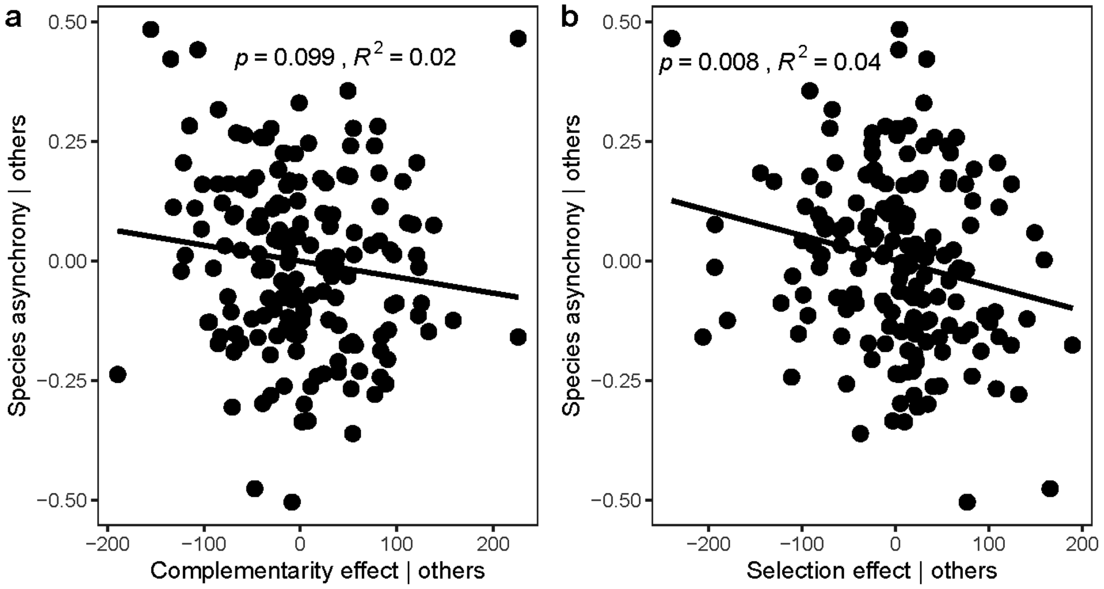

Supplementary Figure 3. Effects of eCO_2 and nitrogen (N) addition on the relationships between (a-c) species asynchrony and complementarity effect (CE), and between (d-f) species asynchrony and selection effect (SE) at each species level. ****P* < 0.001, ***P* < 0.01, **P* < 0.05, .*P* < 0.1. The significant predictors are indicated by text in the figures. Each point represents a plot.

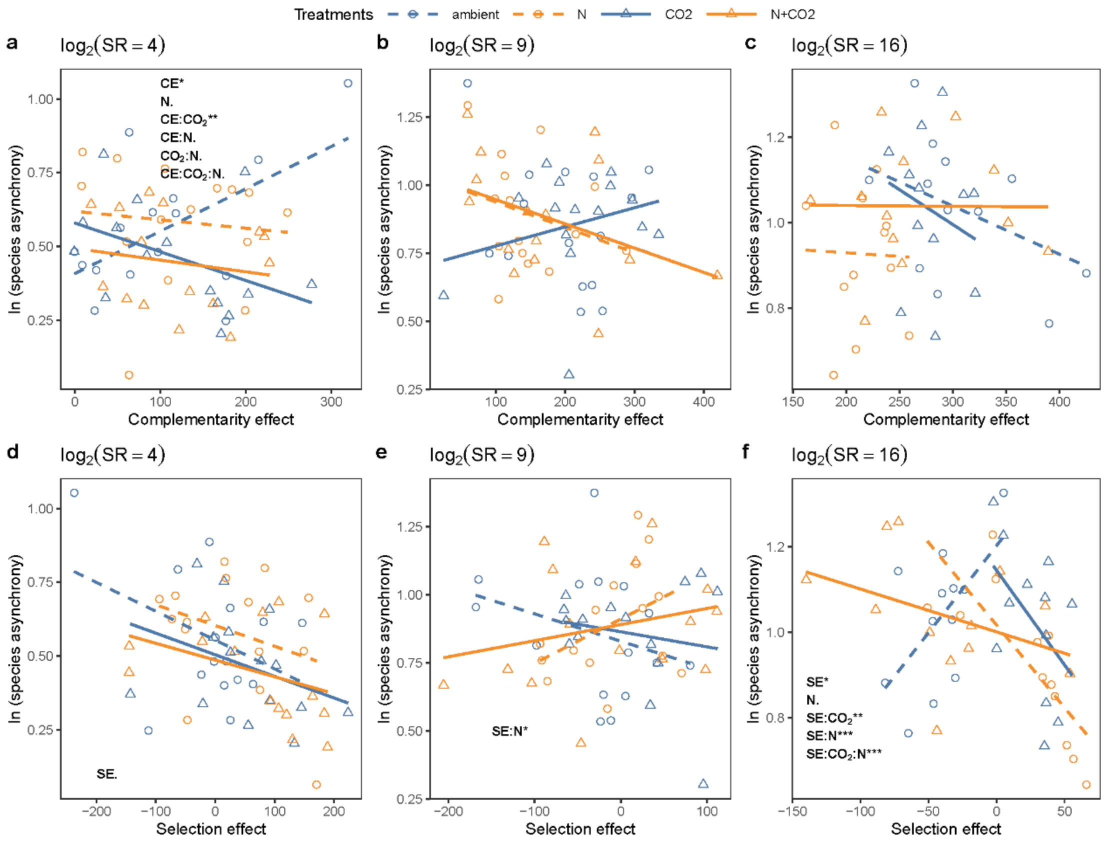

Supplementary Figure 4. Biomass correlation between pairs of the functional groups (C_3 grasses, C_4 grasses, legumes and non-legume forbs) at each treatment level (ambient, eCO_2, N and eCO_2+N).

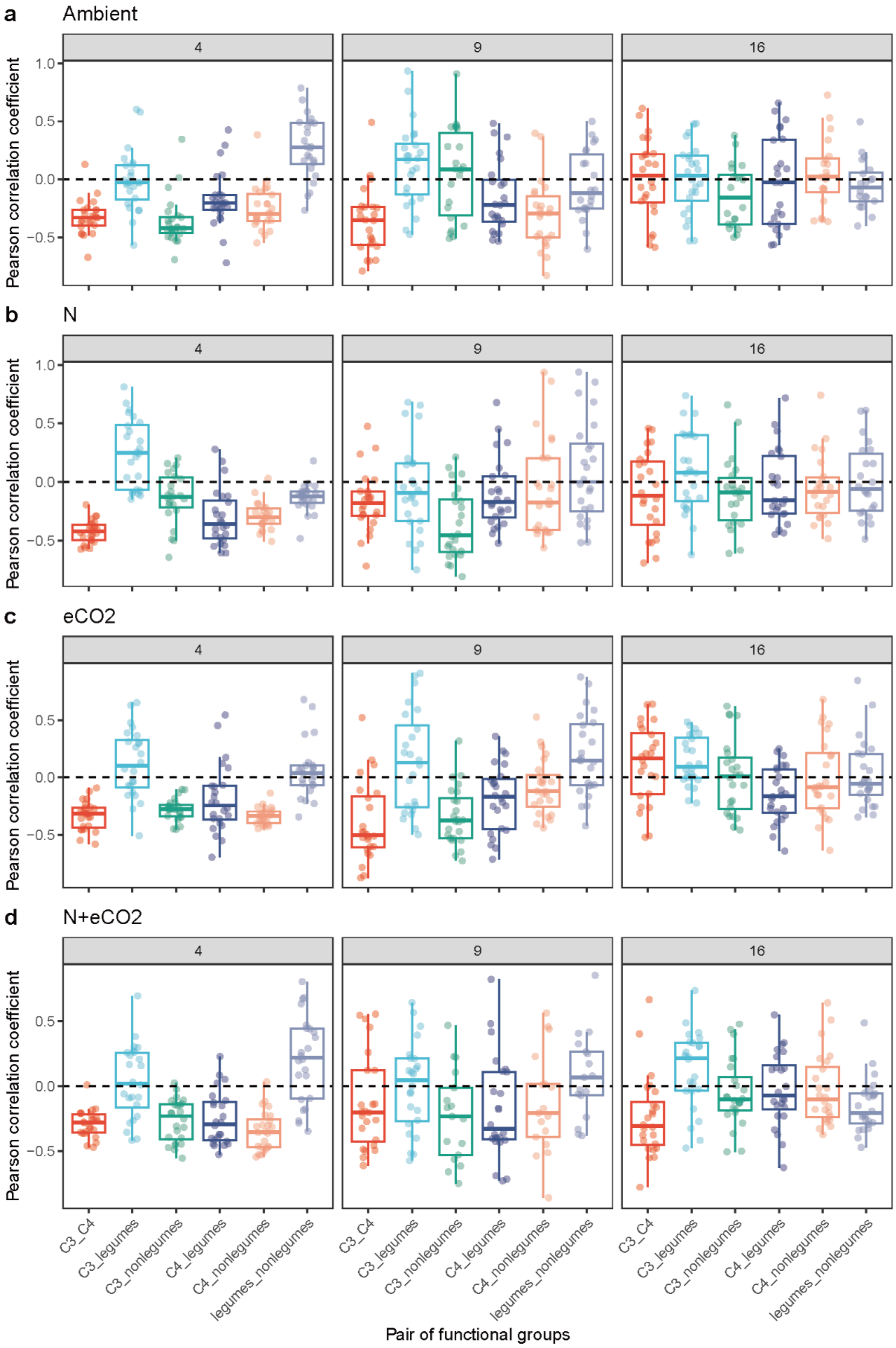

Supplementary Figure 5. Biomass dynamics of each functional group over time under different treatments (ambient, eCO_2, N and eCO_2+N).

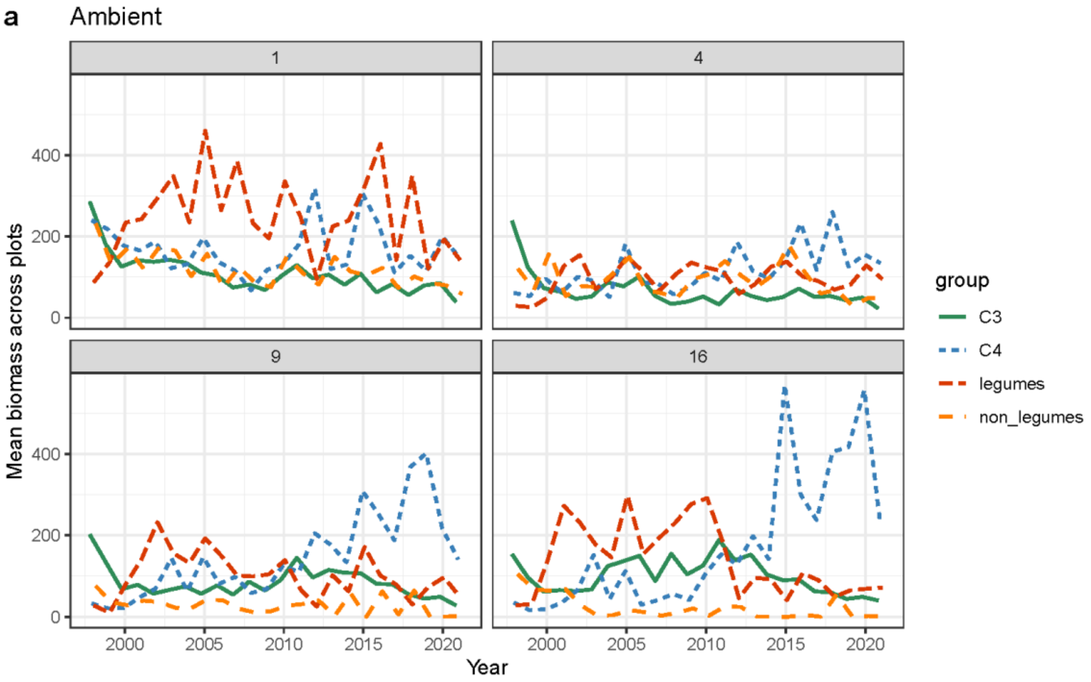

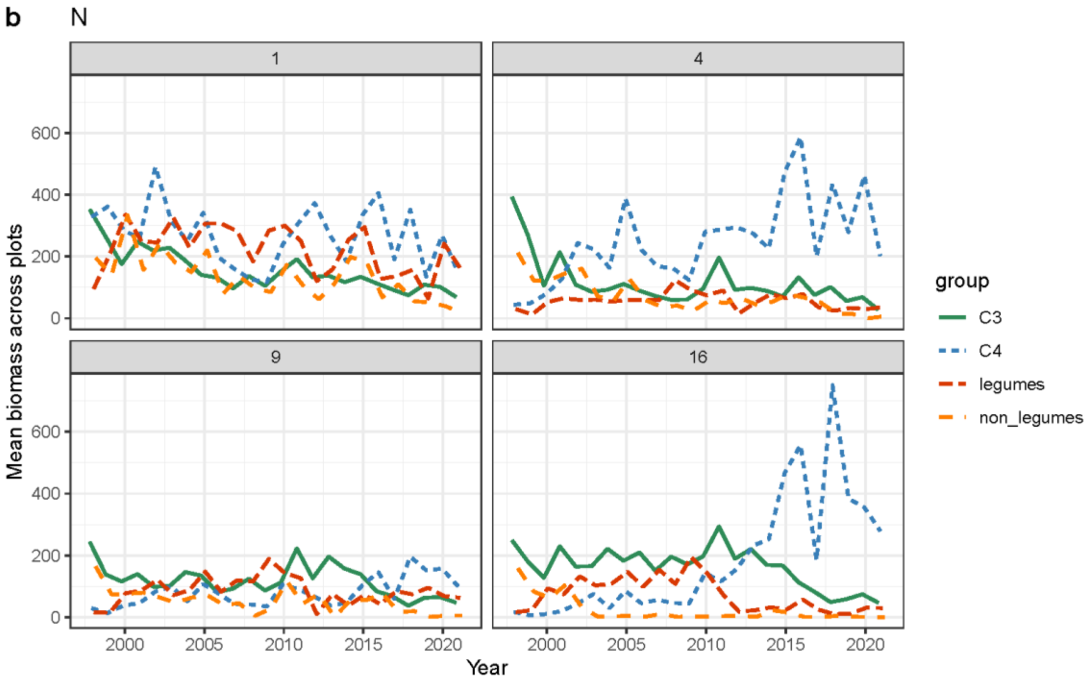

Supplementary Figure 5.

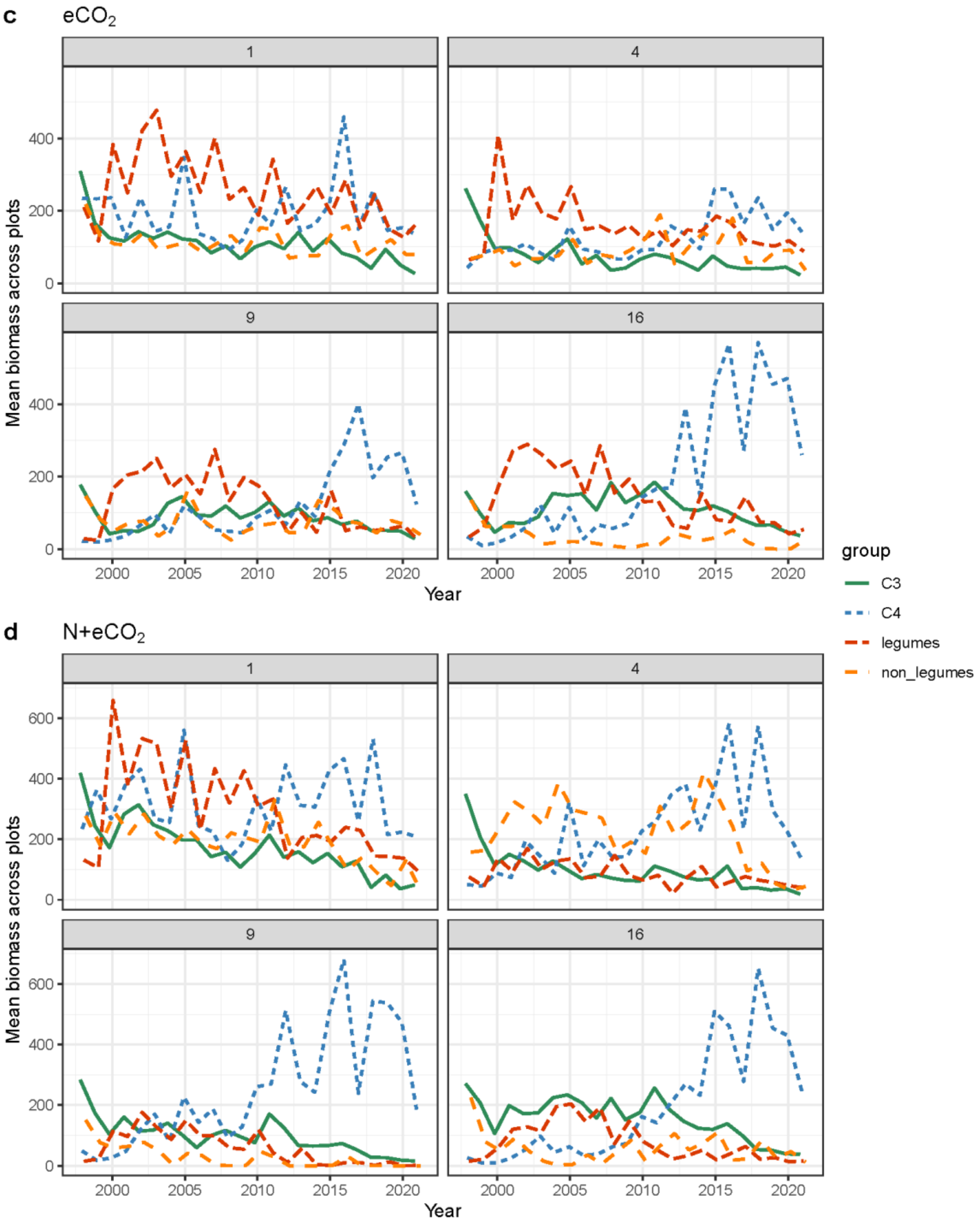

Supplementary Figure 6. Linear mixed-effects models followed by type III ANOVA for the effects of eCO_2 and nitrogen (N) addition on the relationships between species richness (SR) and the biomass of legumes, with year nested in ring as the random intercept. ****P* < 0.001, ***P* < 0.01, **P* < 0.05, .*P* < 0.1, NS: non-significant. The significant predictors are indicated by text in the figures. Each point represents a plot.

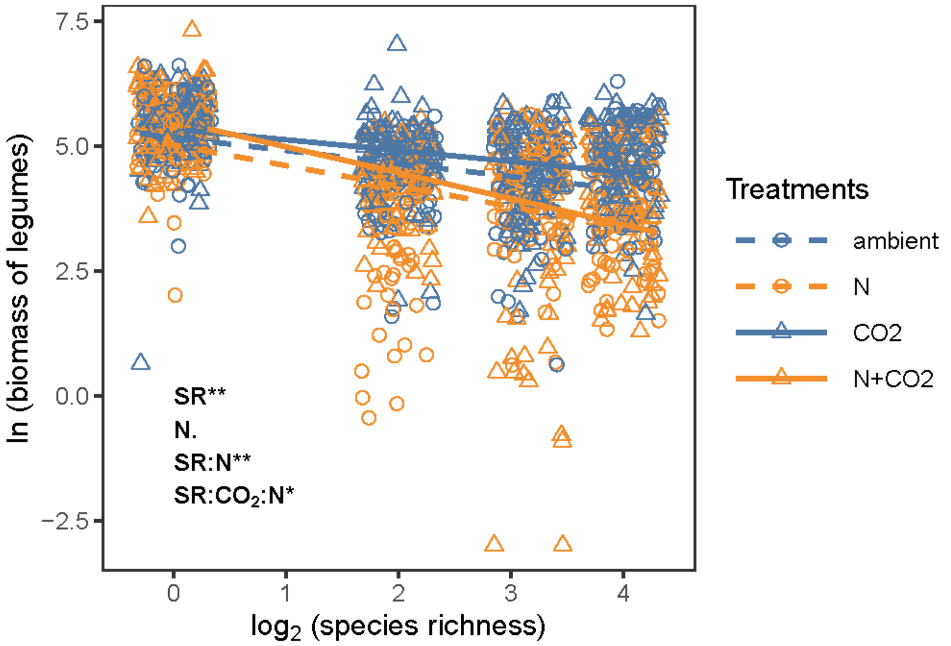

Supplementary Figure 7. Biomass correlation between pairs of the functional groups (C_3 grasses, C_4 grasses, legumes and non-legume forbs) at each species richness level.

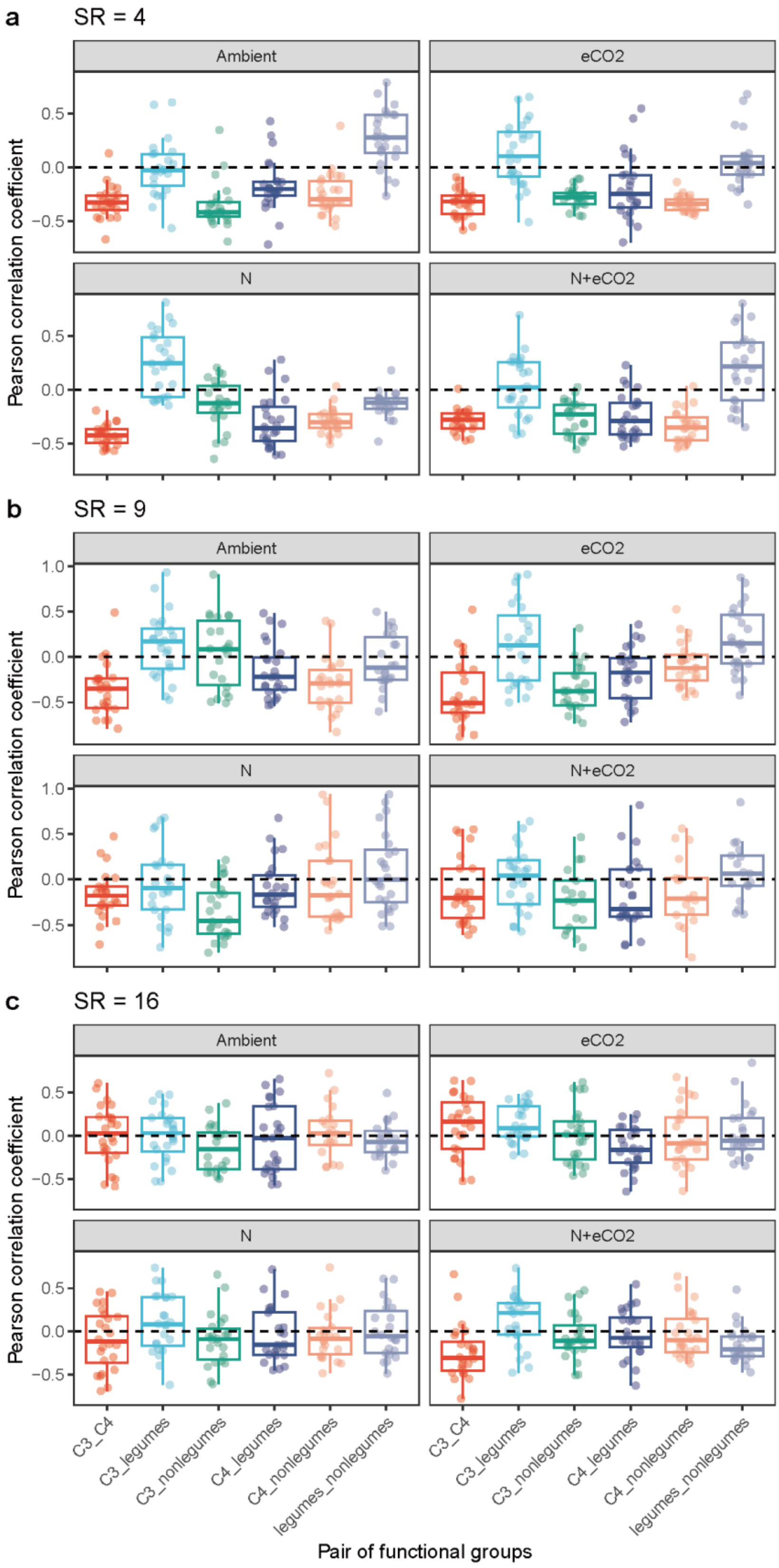

Supplementary Figure 8. Experiment design of BioCON. (a) Aerial view of one ring of the BioCON experiment. The image was sourced from CDR-LTER (cdr-0002) and is used under the CC BY-SA 4.0 license. (b) The number of plots in each ring (rings enriched with CO_2 are in blue), each nitrogen treatment and each species richness level. From 2007, 2 plots in species richness of 9 from each nitrogen treatment in each ring were used for additional precipitation experiments (24 in total). From 2012, another one plot in species richness of 9 from each nitrogen treatment in each ring were used for additional warming experiments (12 in total). (c) The total number of plots in each treatment across years.

a

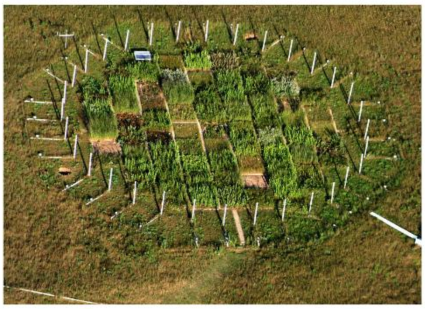

b

Ring ID	Nitrogen	Species richness	Number of plots	Total number of plots	Ring ID	Nitrogen	Species richness	Number of plots	Total number of plots
1	N ambient	1	10	24	4	N ambient	1	11	25
		4	5				4	5	
		9	5				9	5	
		16	4				16	4	
	N addition	1	11	25		N addition	1	10	24
		4	5				4	5	
		9	5				9	5	
		16	4				16	4	
2	N ambient	1	10	24	5	N ambient	1	11	25
		4	5				4	5	
		9	5				9	5	
		16	4				16	4	
	N addition	1	11	25		N addition	1	10	24
		4	5				4	5	
		9	5				9	5	
		16	4				16	4	
3	N ambient	1	11	25	6	N ambient	1	11	25
		4	5				4	5	
		9	5				9	5	
		16	4				16	4	
	N addition	1	11	25		N addition	1	11	25
		4	5				4	5	
		9	5				9	5	
		16	4				16	4	

c

	1998~2006	2007~2011	2012~2021
CO_2amb−Namb	74	68	65
CO_2amb+Nrich	74	68	65
CO_2rich	74	68	65
CO_2rich+Nrich	74	68	65

Supplementary Figure 9. Results of linear models for each species for the relationship between weighted predicted biomass and the observed biomass. Each point represents the data in one plot and one year. The weighted predicted biomass is calculated by multiplying the predicted proportion of biomass from the model for each species by the observed total biomass of the plot. R^2 in the blue excluded species richness of 1, while R^2 in the black included it. Achmil = *Achillea millefolium*, Agrrep = *Agropyron repens*, Amocan = *Amorpha canescens*, Andger = *Andropogon gerardii*, Anecyl = *Anemone cylindrica*, Asctub = *Asclepias tuberosa*, Bougra = *Bouteloua gracilis*, Broine = *Bromus inermis*, Koecri = *Koeleria cristata*, Lescap = *Lespedeza capitata*, Lupper = *Lupinus perennis*, Petvil = *Petalostemum villosum*, Poapra = *Poa pratensis*, Schsco = *Schizachyrium scoparium*, Solrig = *Solidago rigida*, Sornut = *Sorghastrum nutans*.

Supplementary Figure 9.

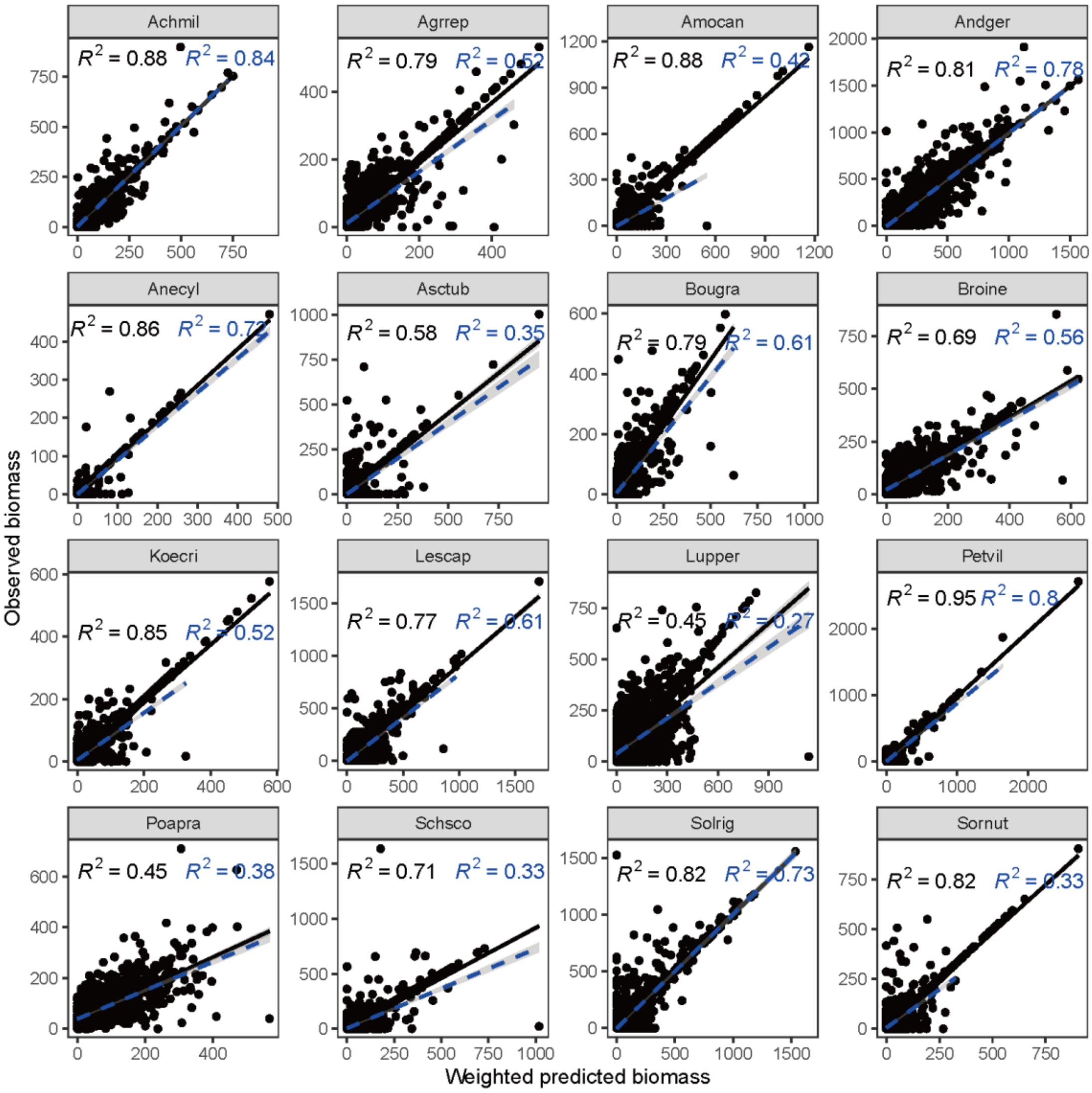

Supplementary Figure 10. Pairwise Pearson correlations between predictor variables. Correlation coefficient ≥ 0.7 are strongly correlated.

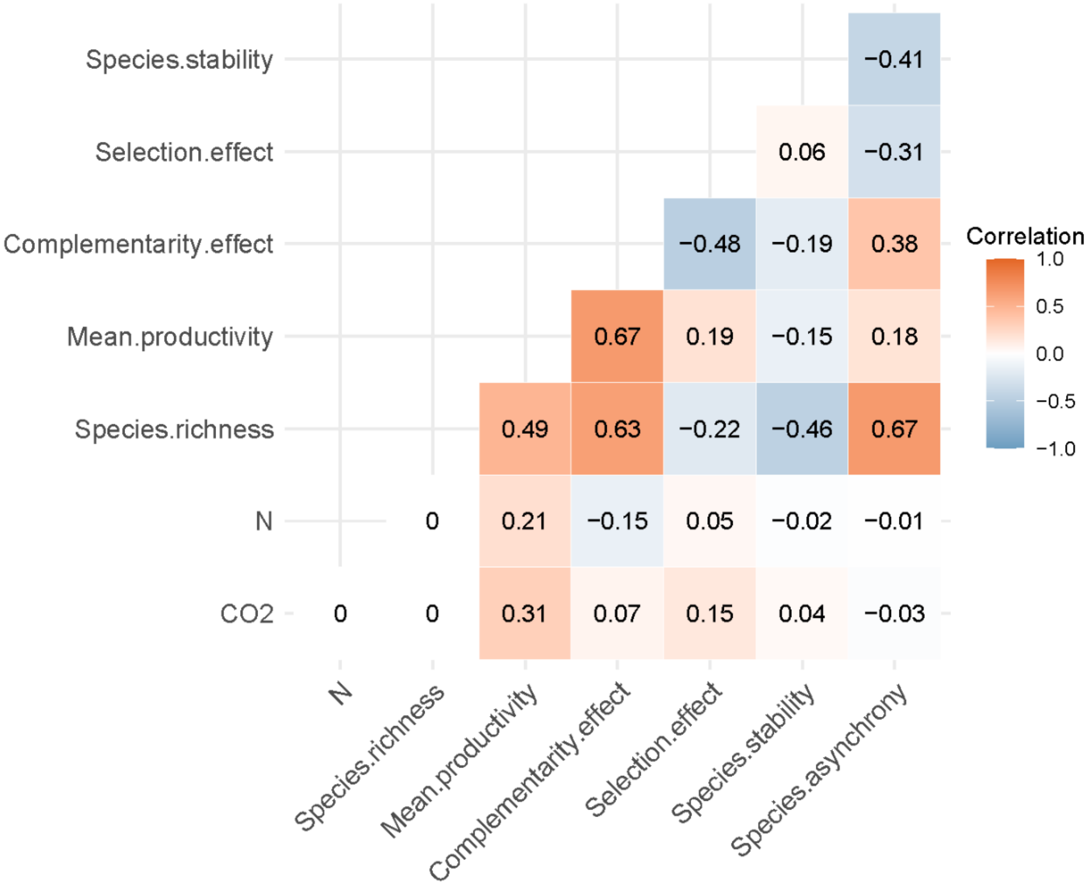

Supplementary Figure 11. Priori overall SEM model to test how species richness affected community stability and its two partitions by affecting biodiversity effects.

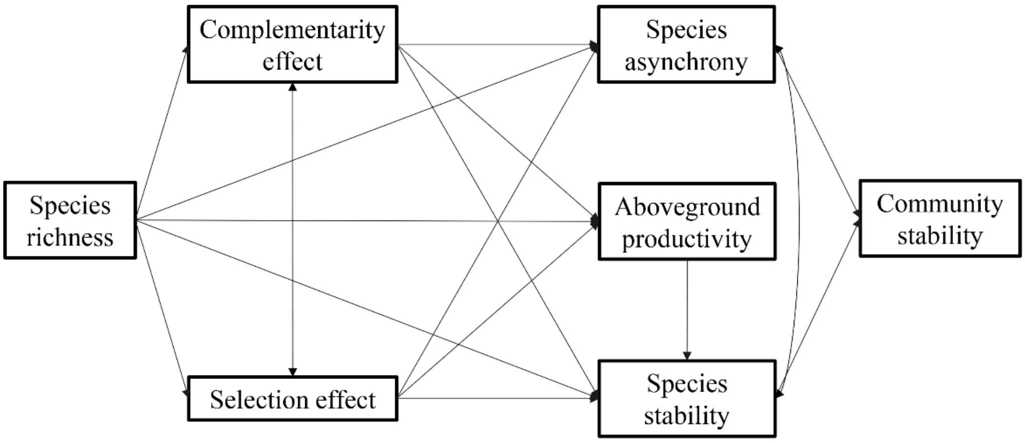

Supplementary Table 1. Model-wide interactions in multi-group structural equation model (Figure 4) showing tests for differences of model paths under different treatments. Model summary statistics of SEM (Fisher's C = 16.112, P = 0.445, AIC = 80.112, d.f. = 16). Stab = community stability; asy = species asynchrony; spp_stab = species stability; CE = complementarity effect; SE = selection effect; p.mean = mean of productivity; SR = species richness; trt = treatment; Estimate = slope coefficient; Std.Error = standard error associated with the (unstandardized slope coefficient); DF = degrees of freedom; Crit.Value = t statistic; Std.Estimate = standardized slope coefficient.

Model-wide interactions

Response	Predictor	Test.Stat	DF	P.Value
**Stab**	**asy:trt**	$2.73*10^{30}$	1	**0.001**
Stab	trt:spp_stab	$2.73*10^{30}$	1	0.416
asy	CE:trt	12.2	1	0.607
asy	trt:SE	12.2	1	0.772
asy	trt:SR	12.2	1	0.118
spp_stab	SR:trt	42.8	1	0.572
**p.mean**	**CE:trt**	2224.2	1	**0.001**
p.mean	trt:SE	2224.2	1	0.895
p.mean	trt:SR	2224.2	1	0.120
**CE**	**SR:trt**	123.3	1	**0.041**
**SE**	**SR:trt**	5.8	1	**0.030**

Ambient

Response	Predictor	Estimate	Std.Error	DF	Crit.Value	P.Value	Std.Estimate
**Stab**	**asy**	1.000	<0.001	37	$7.25*10^{14}$	**<0.001**	1.102
Stab	spp_stab	1.000	<0.001	160	$1.07*10^{15}$	<0.001	0.649
asy	CE	-0.001	<0.001	159	-3.048	0.003	-0.271
asy	SE	-0.001	<0.001	159	-4.169	<0.001	-0.217
asy	SR	0.280	0.023	159	12.425	<0.001	0.805
spp_stab	SR	-0.105	0.016	161	-6.582	<0.001	-0.510
**p.mean**	**CE**	0.002	<0.001	36	28.341	**<0.001**	0.771
p.mean	SE	0.002	<0.001	159	12.731	<0.001	0.370
p.mean	SR	0.052	0.016	159	3.170	0.002	0.126
**CE**	**SR**	102.786	14.448	38	7.114	**<0.001**	0.761
**SE**	**SR**	-18.798	12.405	38	-1.515	**0.138**	-0.224

Supplementary Table 1.

eCO_2							
**Stab**	**asy**	1.000	<0.001	37	$8.44*10^{14}$	**<0.001**	0.979
Stab	spp_stab	1.000	<0.001	160	$1.07*10^{15}$	<0.001	0.740
asy	CE	-0.001	<0.001	159	-3.048	0.003	-0.225
asy	SE	-0.001	<0.001	159	-4.169	<0.001	-0.203
asy	SR	0.280	0.023	159	12.425	<0.001	0.770
spp_stab	SR	-0.105	0.016	161	-6.582	<0.001	-0.380
**p.mean**	**CE**	0.002	<0.001	36	22.463	**<0.001**	0.701
p.mean	SE	0.002	<0.001	159	12.731	<0.001	0.369
p.mean	SR	0.052	0.016	159	3.170	0.002	0.129
**CE**	**SR**	80.582	13.037	38	6.181	**<0.001**	0.690
**SE**	**SR**	-0.486	12.324	38	-0.040	**0.969**	-0.006
N							
**Stab**	**asy**	1.000	<0.001	37	$7.8*10^{14}$	**<0.001**	1.128
Stab	spp_stab	1.000	<0.001	160	$1.07*10^{15}$	<0.001	0.865
asy	CE	-0.001	<0.001	159	-3.048	0.003	-0.196
asy	SE	-0.001	<0.001	159	-4.169	<0.001	-0.238
asy	SR	0.280	0.023	159	12.425	<0.001	0.920
spp_stab	SR	-0.105	0.016	161	-6.582	<0.001	-0.448
**p.mean**	**CE**	0.003	<0.001	36	25.342	<0.001	0.864
p.mean	SE	0.002	<0.001	159	12.731	**<0.001**	0.496
p.mean	SR	0.052	0.016	159	3.170	0.002	0.176
**CE**	**SR**	52.994	11.322	38	4.681	**<0.001**	0.622
**SE**	**SR**	10.183	11.765	38	0.866	**0.392**	0.126
eCO_2+N							
**Stab**	**asy**	1.000	<0.001	37	$1.04*10^{15}$	**<0.001**	1.053
Stab	spp_stab	1.000	<0.001	160	$1.07*10^{15}$	<0.001	0.621
asy	CE	-0.001	<0.001	159	-3.048	0.003	-0.224
asy	SE	-0.001	<0.001	159	-4.169	<0.001	-0.294
asy	SR	0.280	0.023	159	12.425	<0.001	0.740
spp_stab	SR	-0.105	0.016	161	-6.582	<0.001	-0.468
**p.mean**	**CE**	0.002	<0.001	36	23.429	**<0.001**	1.041
p.mean	SE	0.002	<0.001	159	12.731	<0.001	0.847
p.mean	SR	0.052	0.016	159	3.170	0.002	0.196
**CE**	**SR**	69.893	15.411	38	4.535	**<0.001**	0.576
**SE**	**SR**	-50.926	16.834	38	-3.025	**0.004**	-0.411

Bold values indicate statistically significant effects at $P < 0.1$.

Supplementary Table 2. Statistics results of linear mixed-effects models using Anova function (with type III SS) for the effect of eCO_2, N addition and complementarity effect (CE) or selection effect (SE) on mean of productivity (mean_prod), species asynchrony or species stability, and the effect of eCO_2, nitrogen (N) addition and species asynchrony (asy) or species stability (spp_stab) on community stability across 24 years. Community stability, species asynchrony and species stability were logarithm transformed. All tests were two-sided.

Predictors	Mean of productivity			Community stability			Species asynchrony			Species stability		
	χ^2	df	*P* value	χ^2	df	*P* value	χ^2	df	*P* value	χ^2	df	*P* value
CE	129.2	1	**<0.001**				1.52	1	0.218	0.62	1	0.430
CO_2	0.28	1	0.599				0.28	1	0.600	<0.01	1	0.954
N	0.14	1	0.706				1.85	1	0.174	1.65	1	0.199
CE:CO_2	1.55	1	0.212				1.18	1	0.278	0.23	1	0.633
CE:N	4.97	1	**0.025**				2.28	1	0.131	2.89	1	**0.089**
CO_2:N	10.08	1	**0.001**				0.54	1	0.462	0.02	1	0.891
CE:CO_2:N	20.00	1	**<0.001**				0.80	1	0.370	0.49	1	0.485
SE	2.65	1	0.103				4.16	1	**0.041**	0.58	1	0.447
CO_2	7.84	1	**0.005**				0.01	1	0.906	0.50	1	0.479
N	0.42	1	0.518				0.19	1	0.667	0.16	1	0.690
SE:CO_2	3.93	1	**0.048**				0.15	1	0.696	3.17	1	**0.075**
SE:N	12.81	1	<0.001				0.88	1	0.348	0.45	1	0.503
CO_2:N	0.17	1	0.680				0.25	1	0.614	1.35	1	0.246
SE:CO_2:N	9.25	1	**0.002**				0.09	1	0.769	1.00	1	0.317
asy				60.45	1	**<0.001**						
CO_2				0.06	1	0.813						
N				1.50	1	0.220						
asy:CO_2				<0.01	1	0.986						
asy:N				1.27	1	0.259						
CO_2:N				1.84	1	0.175						
asy:CO_2:N				1.26	1	0.261						
spp_stab				0.69	1	0.405						
CO_2				0.01	1	0.911						

Supplementary Table 2.

N	0.10	1	0.748
spp_stab:CO_2	0.74	1	0.391
spp_stab:N	0.23	1	0.634
CO_2:N	0.24	1	0.621
spp_stab:CO_2:N	0.49	1	0.486
mean_prod	0.26	1	0.613
CO_2	0.74	1	0.388
N	0.38	1	0.536
mean_prod:CO_2	0.74	1	0.390
mean_prod:N	0.37	1	0.543
CO_2:N	<0.01	1	0.987
mean_prod:CO_2:N	<0.01	1	0.987

Bold values indicate statistically significant effects at P < 0.1.

Supplementary Note 1: sensitivity analyses

To fill the missing values in biomass data, we estimated the species-level biomass for the affected plots and years using species cover data. We fitted linear models to establish relationships between biomass and cover for each species, and then predicted biomass using these equations. However, the biomass prediction models do not perform well for some species (low R^2 for the relationship between predicted and measured biomass). To evaluate whether our main findings are sensitive to these modeling choices, we conducted several sensitivity analyses and interpreted the outcomes as follows:

1. we compared different types of models.
2. we compared the results from data including and excluding predicted biomass.
3. we added a variability to the predicted biomass to show how stable the results are.
4. we tested the proportion of predicted biomass within the total dataset.
5. we interpreted why some species showed low predictive accuracy.

Overall, the sensitivity analyses demonstrated that our results are robust to these modeling decisions.

1. Comparing different types of models

In addition to linear relationship, we also considered exponential and power function relationships between species biomass (y) and observed cover (x) across years in each plot. Based on these relationships, we considered 5 models, including:

$$\begin{aligned}
&\text{Model 1:} \quad y = ax \\
&\text{Model 2:} \quad \ln y = ax \\
&\text{Model 3:} \quad \ln y = a \ln x \\
&\text{Model 4:} \quad y = a\left(e^{bx} - 1\right) \\
&\text{Model 5:} \quad y = ax^b
\end{aligned}$$

where **Model 1-3** were fitted by linear models using the `lm` function. **Model 4-5** were fitted by non-linear models using the `nlsLM` function from the `minpack.lm` package. Here are the codes for model fits.

```r
# zero-intercept regression by mean value across years of each species
slp_r2 = sp.mean %>%
  group_by(spid) %>%
  summarise(
    lm_m = list(lm(sp.mean.bio ~ 0 + sp.mean.cover)),
    lm_exp = list(lm(log(sp.mean.bio + 1) ~ 0 + sp.mean.cover)),
    lm_p = list(lm(log(sp.mean.bio + 1) ~ 0 + log(sp.mean.cover + 1))),
    nls_exp = list(nlsLM(sp.mean.bio ~ a * (exp(b * sp.mean.cover) - 1),
                         start = list(a = 1, b = 0.1),
                         control = nls.control(maxiter = 200))),
    nls_p = list(nlsLM(sp.mean.bio ~ a * sp.mean.cover^b, start = list(a = 1, b = 0.1)))
    )
```

We evaluated the linear relationships between the weighted predicted biomass (predicted proportion of each species' biomass multiplied the observed total biomass of the plot) and the observed biomass of each species of each model considered. These relationships were evaluated both across all species richness levels (shown in black) and excluding monocultures (species richness > 1, shown in blue). Most species—with the dataset across all species richness levels (shown in black)—showed good model fits ($R^2 > 0.7$). The corresponding figures for each model are presented below, in the same order as the models described.

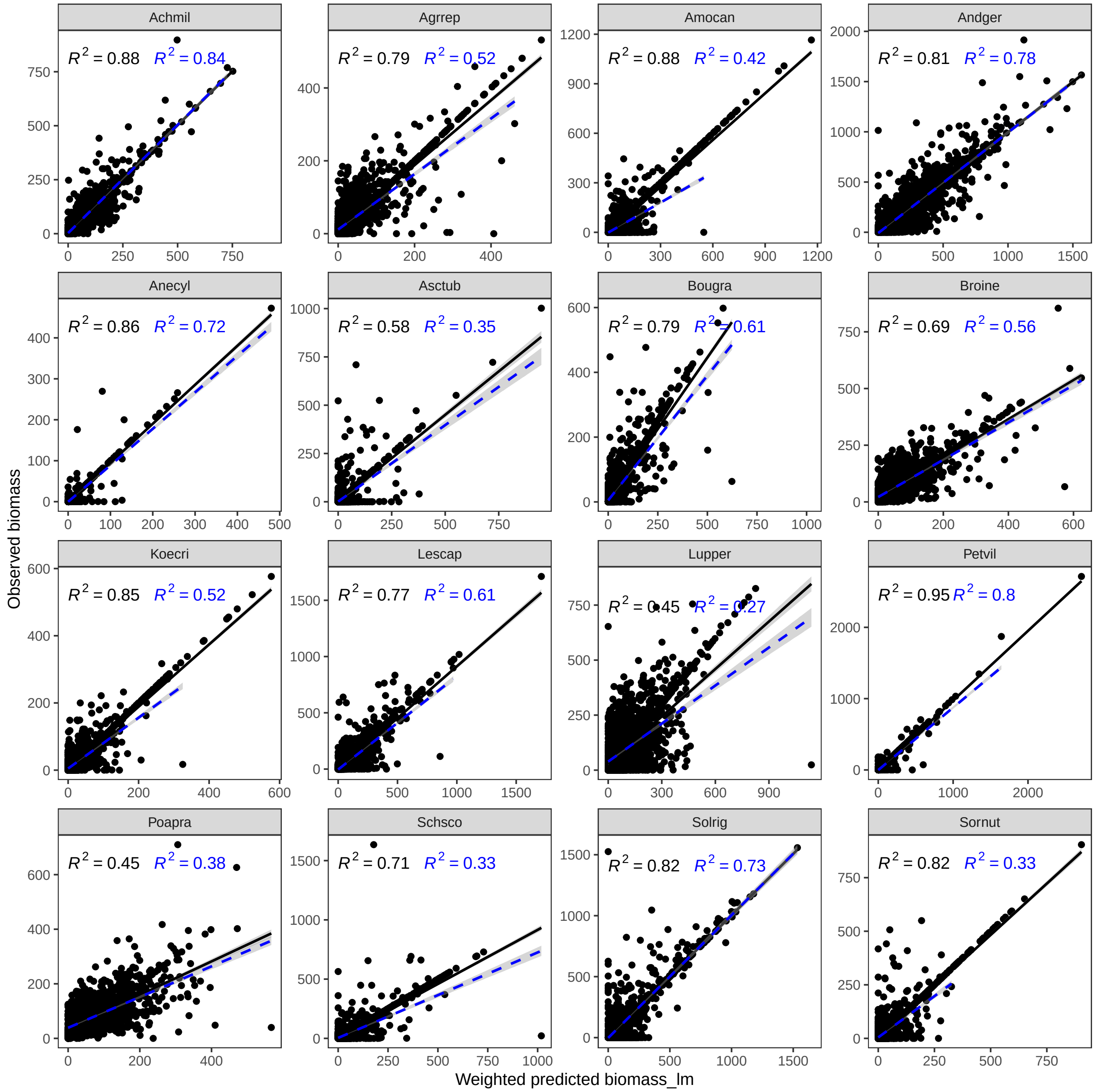

Supplementary Figure 12. The relationship between predicted weighted biomass based on Model 1 and observed biomass of each species.

The regressions are what we had in Supplementary Figure 9.

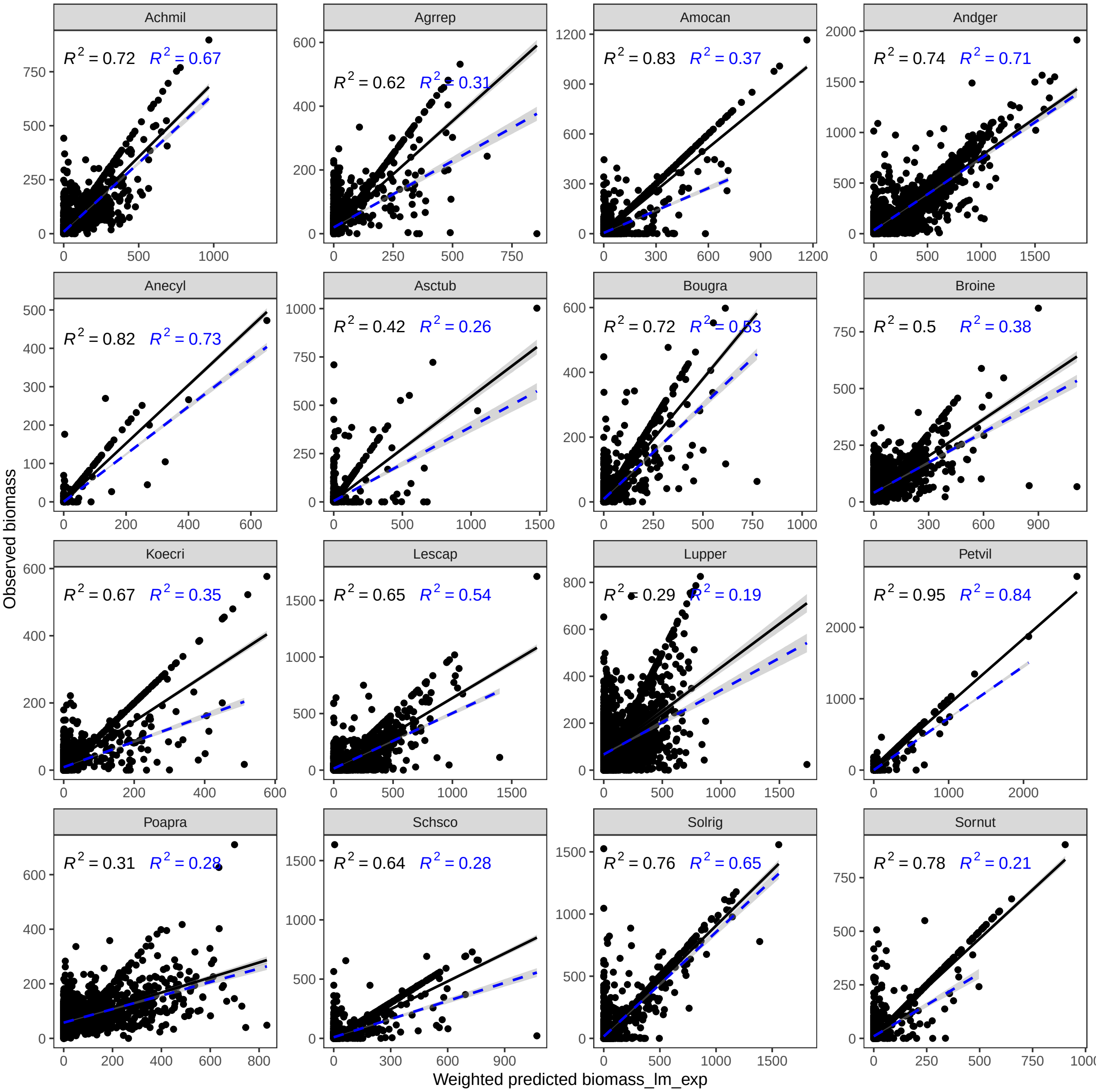

Supplementary Figure 13. The relationship between predicted weighted biomass based on Model 2 and observed biomass of each species.

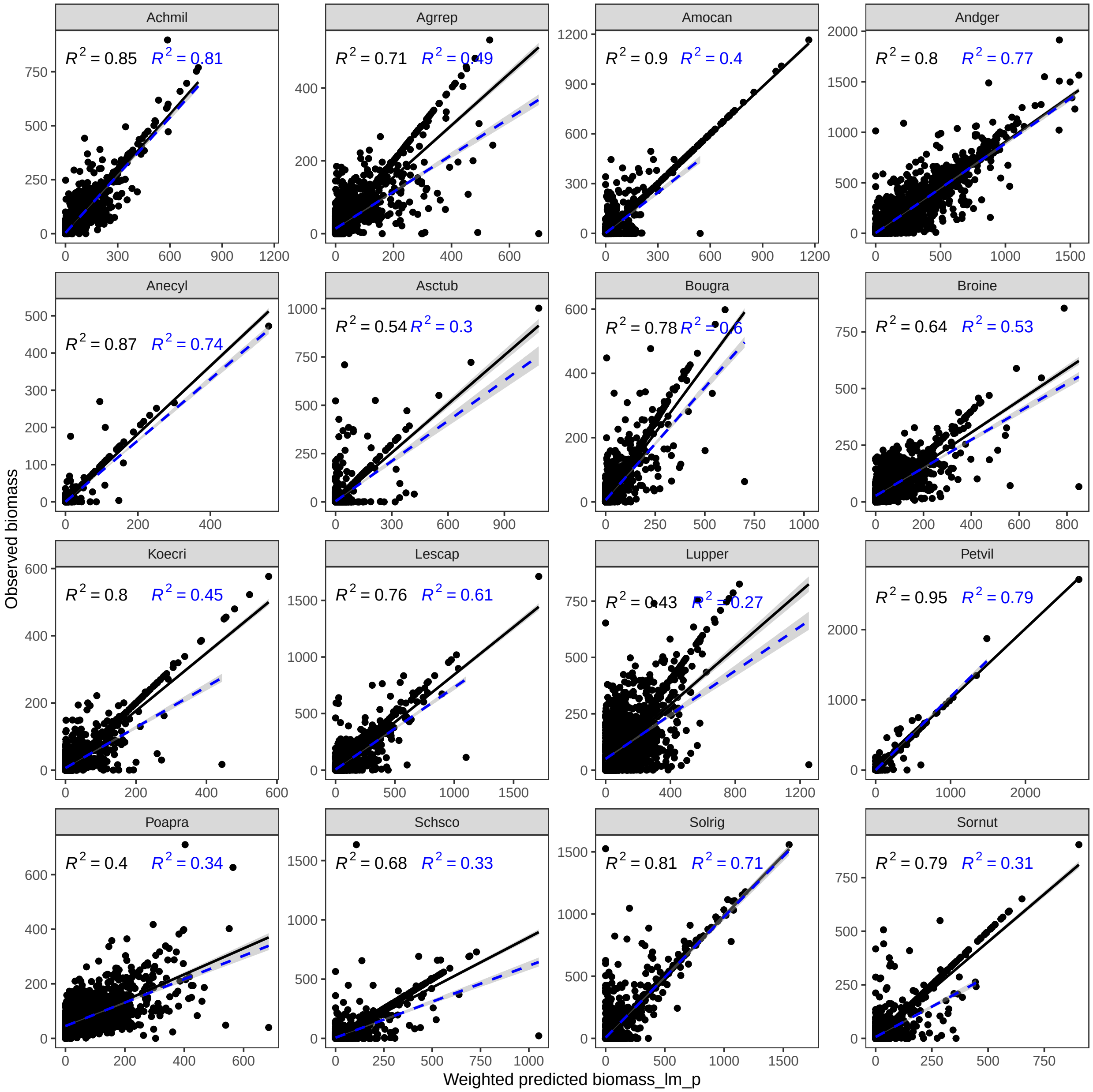

Supplementary Figure 14. The relationship between predicted weighted biomass based on Model 3 and observed biomass of each species.

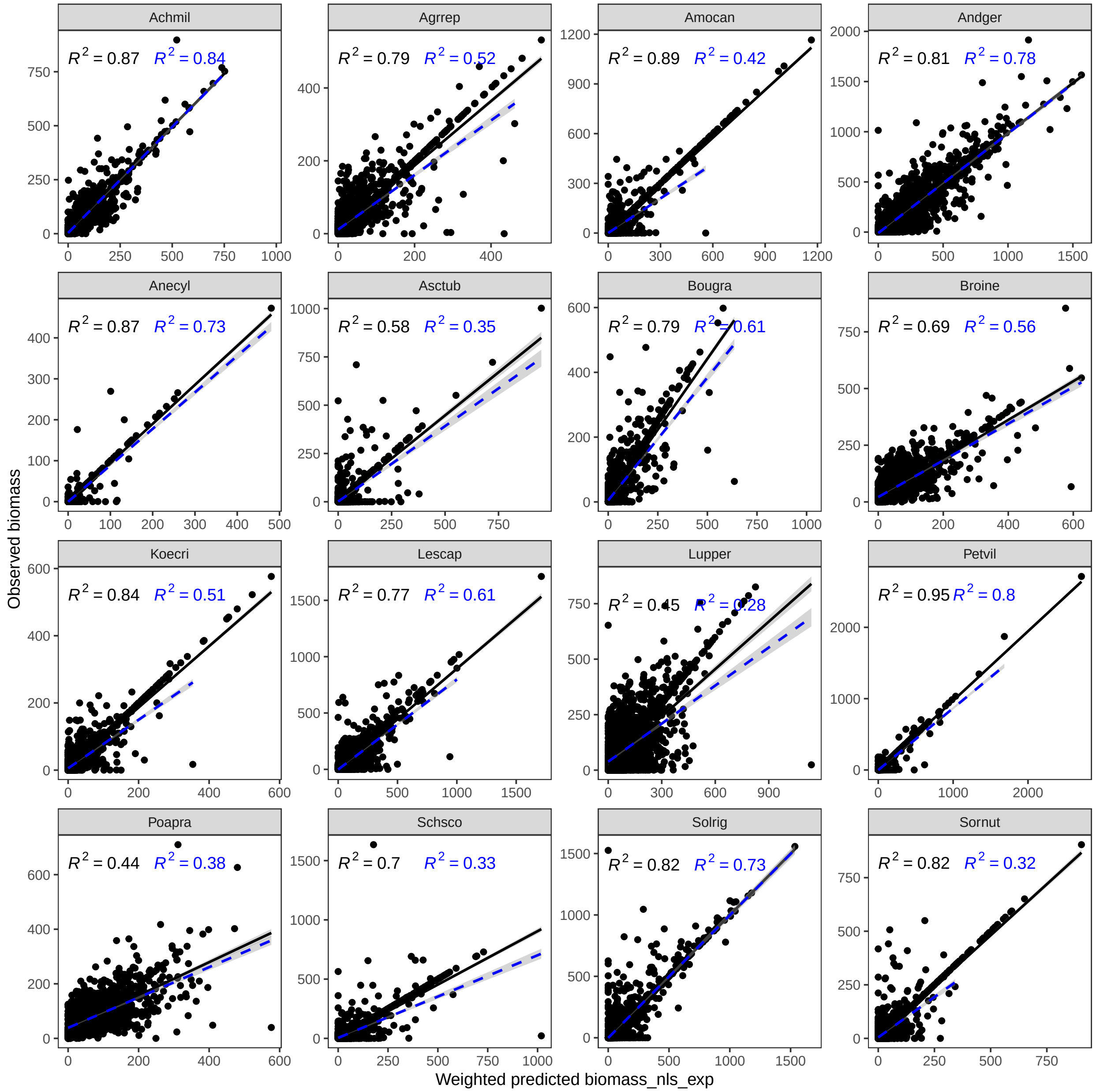

Supplementary Figure 15. The relationship between predicted weighted biomass based on Model 4 and observed biomass of each species.

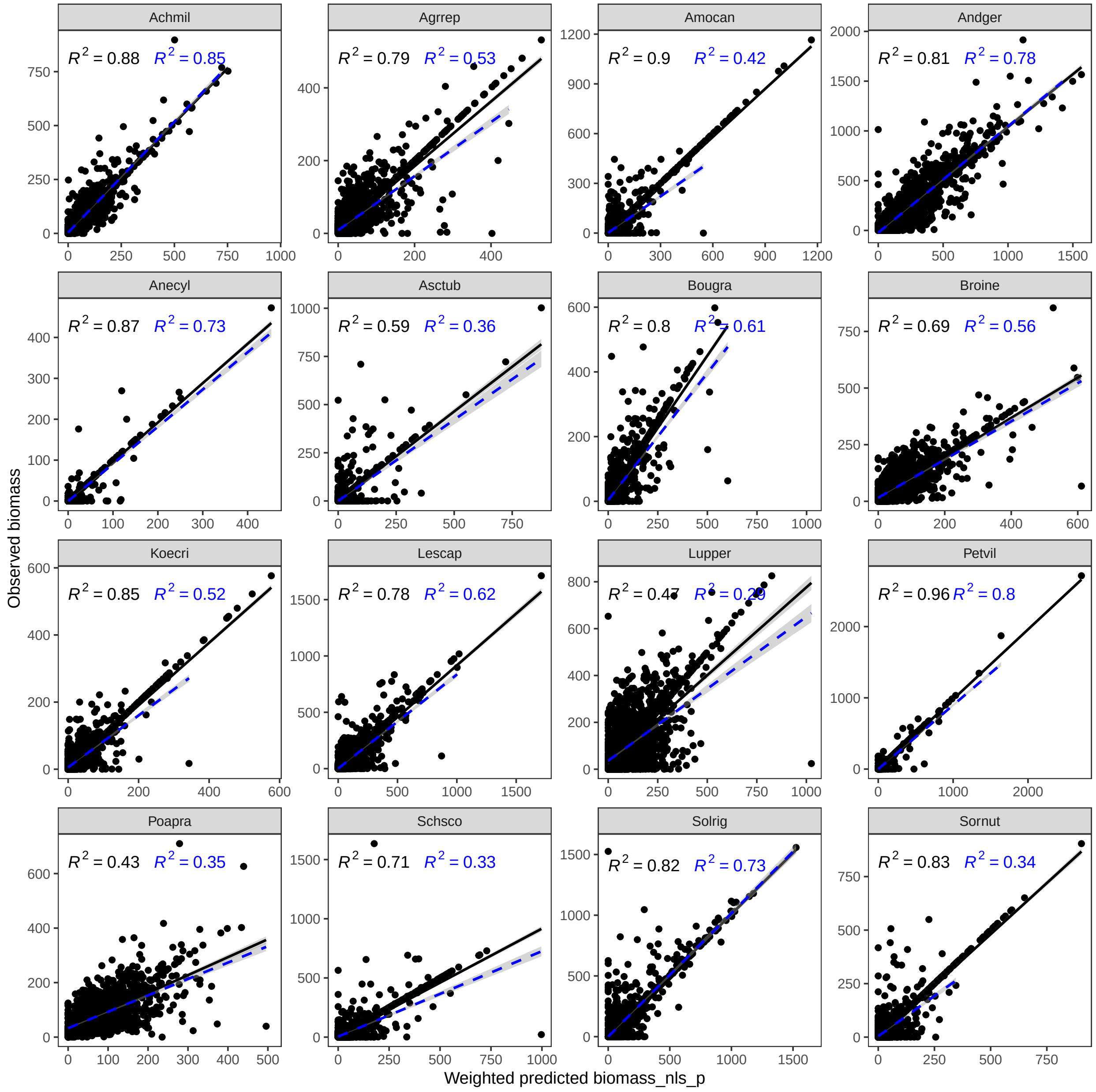

Supplementary Figure 16. The relationship between predicted weighted biomass based on Model 5 and observed biomass of each species.

From Supplementary Figure 12-16, we observe that the predictions from different models are are generally similar. Some species have a larger R^2 predicted by models other than the linear model, but these improvements are minimal, e.g, Agrrep ($R^2 = 0.52$ from Model 1 VS. 0.53 from Model 5). Since the prediction for many species from linear models have the highest R^2 and there is no significant d ifferent am ong di fferent mod els, we kep t our original linear regression (Model 1).

2. Comparing the results from data including and excluding predicted biomass.

We used the observed biomass of each species we have (excluded the predicted biomass) to repeat the analyses in Fig. 1-4 in the text.

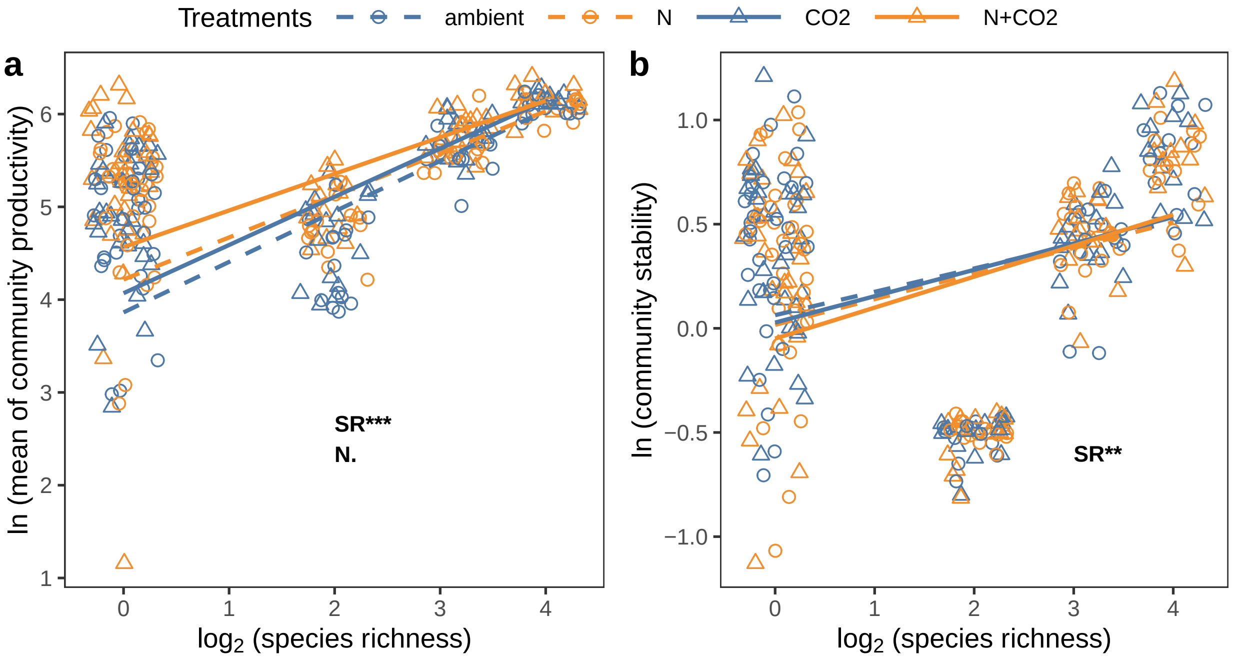

Supplementary Figure 17. Effects of CO2 enrichment and nitrogen (N) addition on species richness-productivity and species richness-stability relationships.

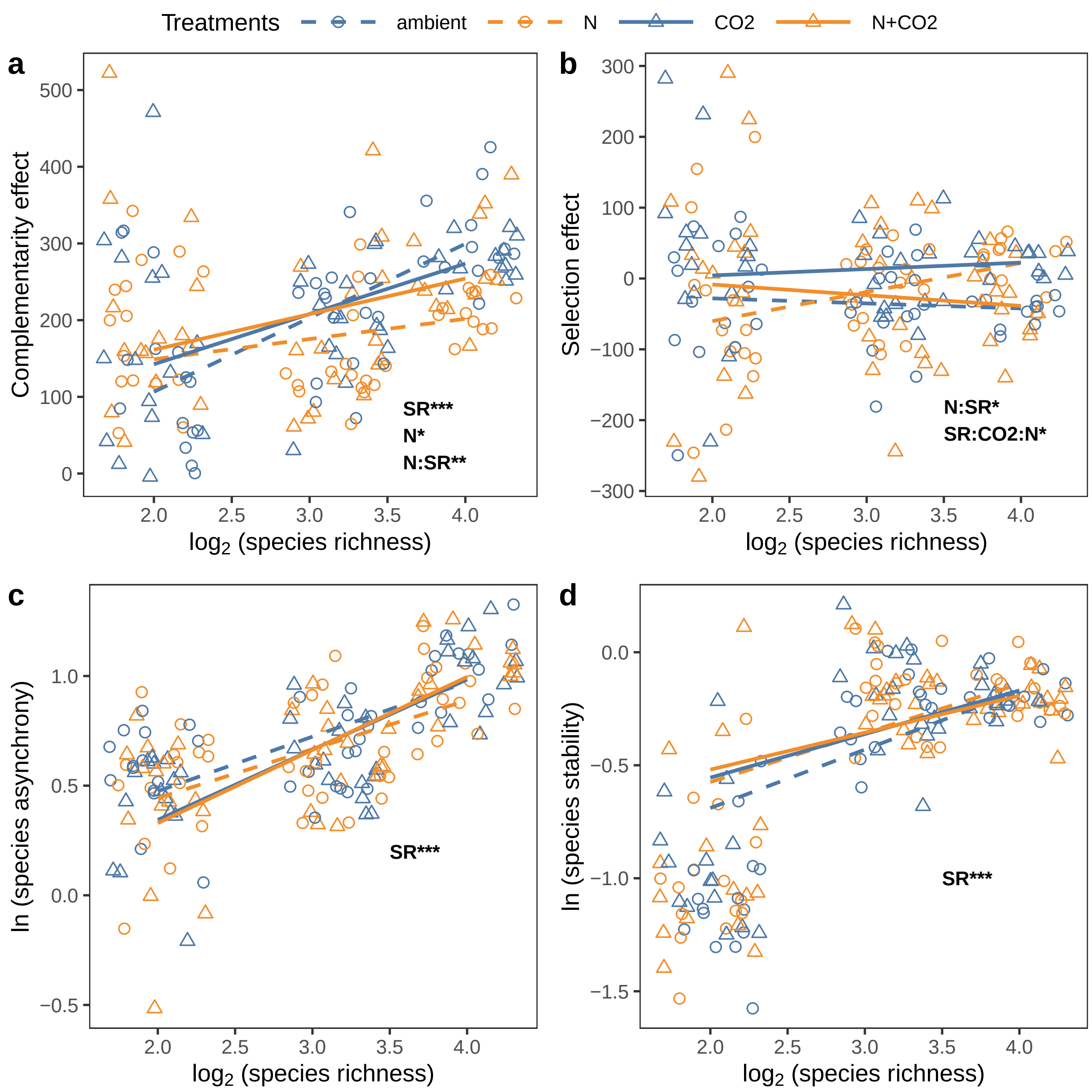

Supplementary Figure 18. Effects of CO2 enrichment and nitrogen (N) addition on the processes underlying species richness-productivity and species richness-stability relationships.

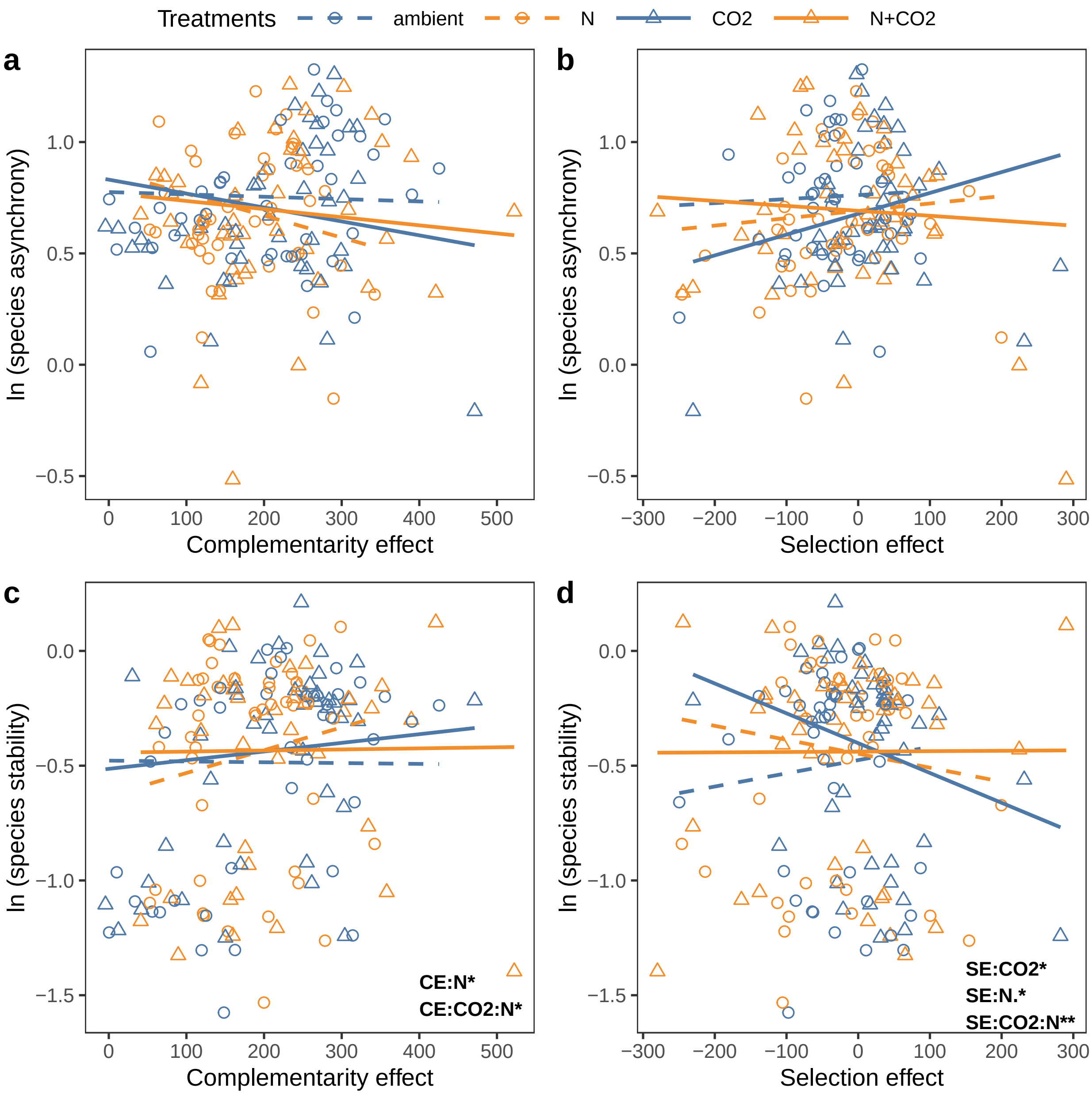

Supplementary Figure 19. Effects of CO2 enrichment and nitrogen (N) addition on the links between species richness-productivity and species richness-stability relationships.

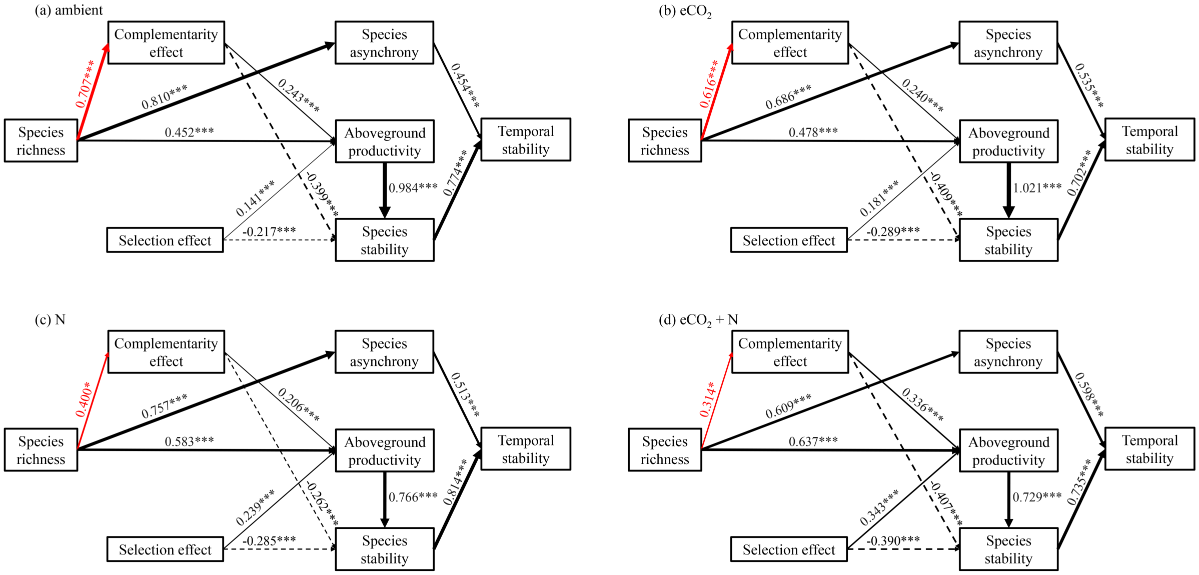

Supplementary Figure 20. Linking biodiversity effects and stability under different treatments.

We observe that, despite some variability, the overall patterns align with those shown in Figs. 1–4 in the main text.The structural equation model (SEM) still supports the conclusion that global change weakens the relationship between species richness and complementary effect, while having no significant impact on the relationships between biodiversity–ecosystem functioning (BEF) and biodiversity–ecosystem stability (BEFS).

3. Adding a variability to the predicted biomass to show how stable the results are

To incorporate random errors into each predicted value, we generated random numbers from a normal distribution, with error magnitudes based on the standard errors of the predictions (`se.fit`). We repeated this simulation for 100 times, and used the average value for the subsequent analyses. Note: we ran it separately because it took a long time.

```
# lm_var <- data.coef %>%
#   select(-(lm_exp:nls_p)) %>%
#   rename(sp.mean.cover = cover, sp.mean.bio = biomass)
#
# set.seed(123)
# n_simulations <- 100
#
# lm_simulations <- vector()
#
```

```
# for (i in 1:nrow(lm_var)) {
# model <- lm_var$lm_m[[i]]
# cover_value <- lm_var$sp.mean.cover[i]
#
# sim_results <- replicate(n_simulations, {
#   pred <- predict(model, newdata = data.frame(sp.mean.cover = cover_value),
#                   se.fit = TRUE)
#   pred$fit + rnorm(length(pred$fit), mean = 0, sd = pred$se.fit)
# })
#
# mean_sim <- mean(sim_results, na.rm = T)
#
# lm_simulations[i] <- sim_results
# }
#
# lm_var$pre_var <- lm_simulations
```

The results for Fig. 1-4 are as follows:

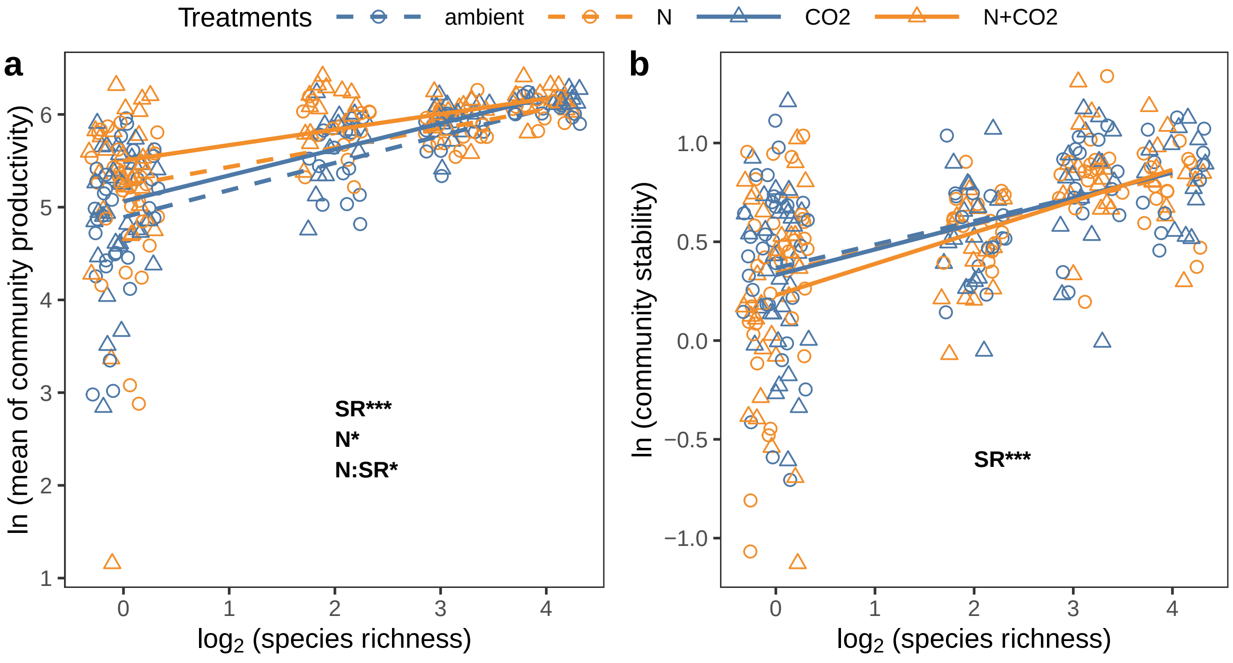

Supplementary Figure 21. Effects of CO2 enrichment and nitrogen (N) addition on species richness-productivity and species richness-stability relationships.

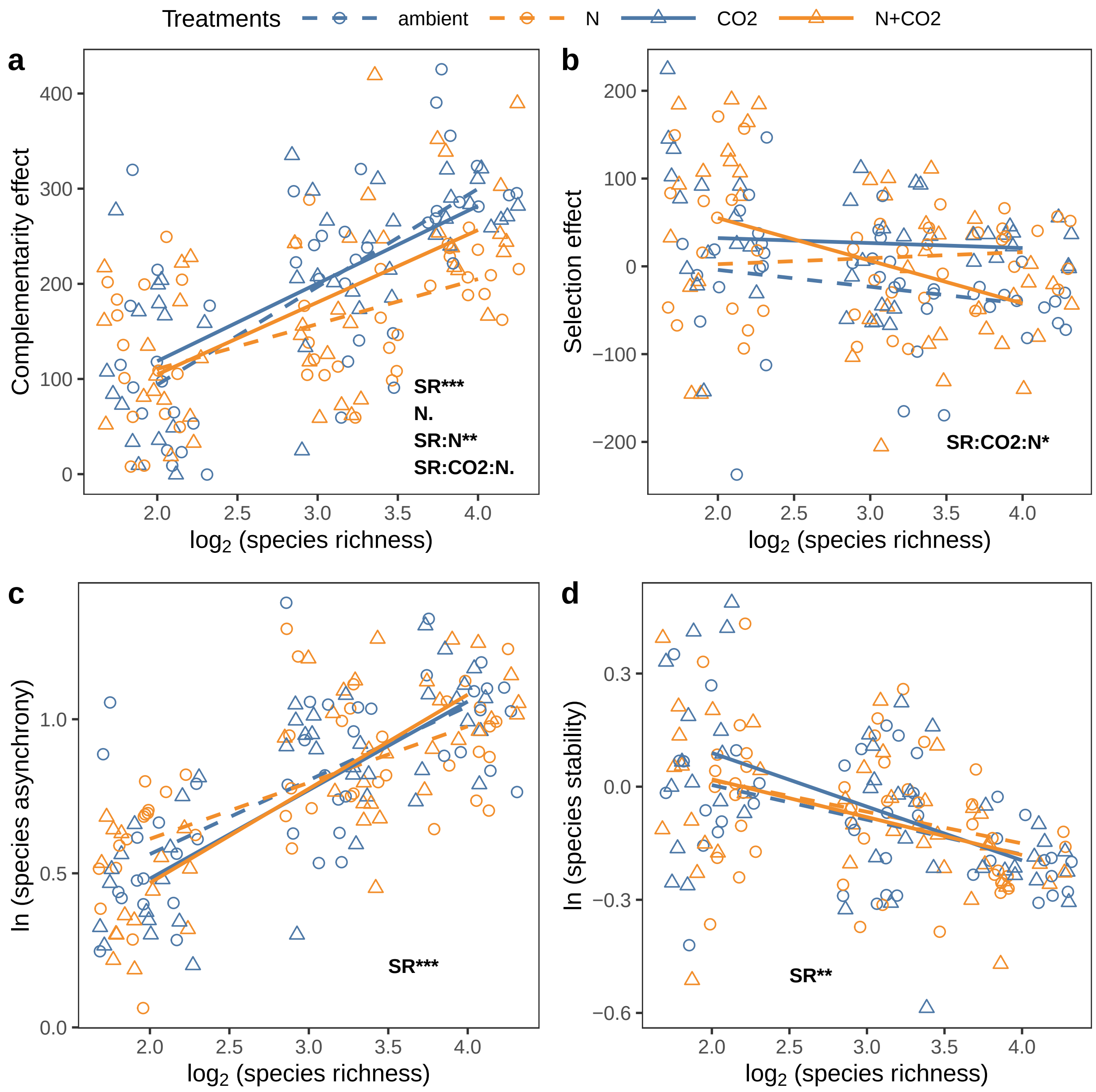

Supplementary Figure 22. Effects of CO2 enrichment and nitrogen (N) addition on the processes underlying species richness-productivity and species richness-stability relationships

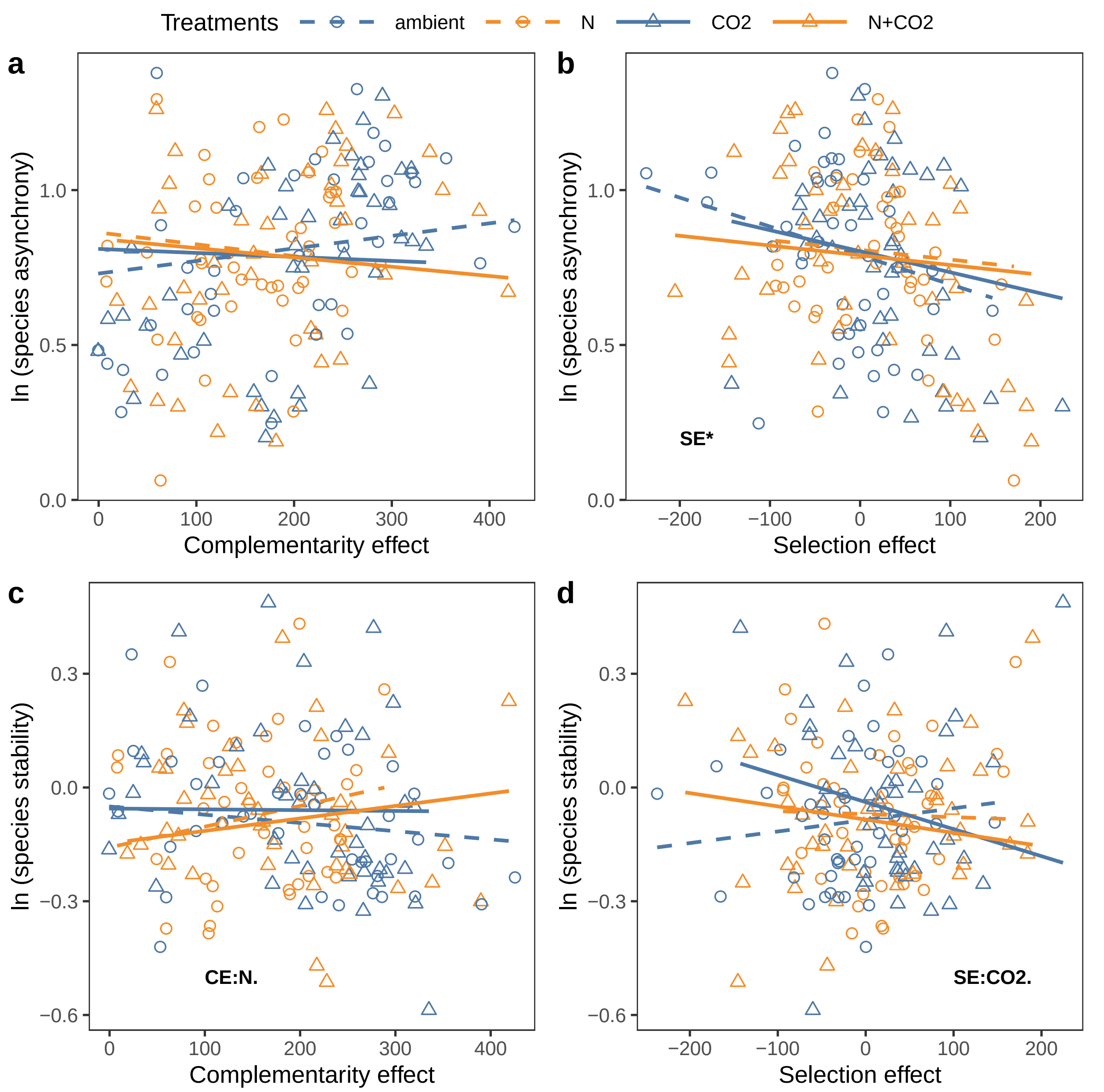

Supplementary Figure 23. Effects of CO2 enrichment and nitrogen (N) addition on the links between species richness-productivity and species richness-stability relationships

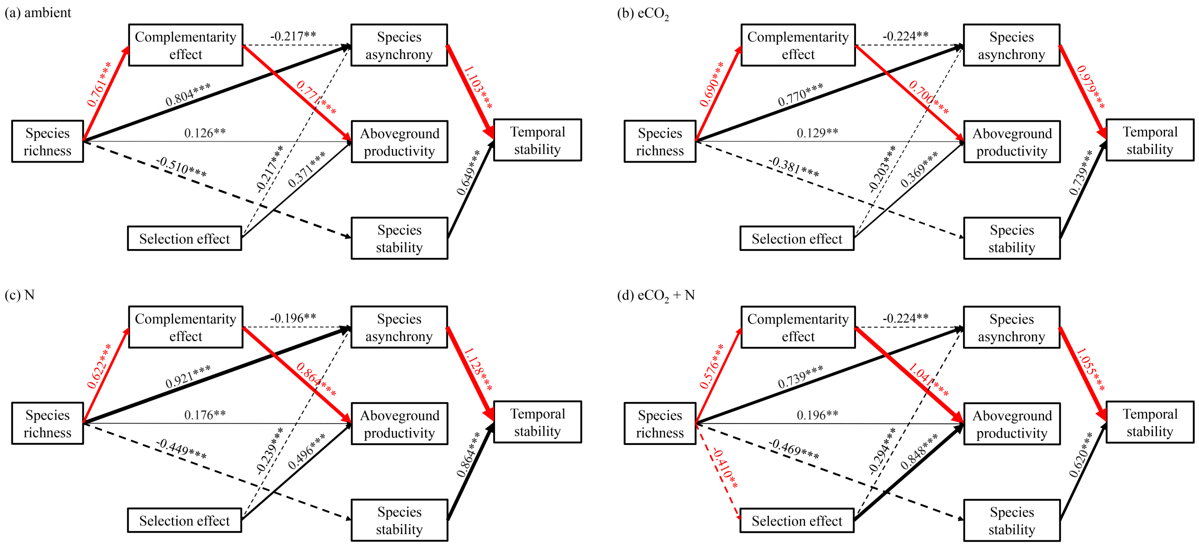

Supplementary Figure 24. Linking biodiversity effects and stability under different treatments.

The results are very similar to what we had in the main text, which suggested that our results are quite robust.

4. The proportion of predicted biomass within the total dataset.

```
# filled NA in biomass with weighed predicted biomass
pre_fill = pre_totbio %>%
  mutate(bio_filled = if_else(is.na(biomass), weighted_lm, biomass))

# select main experiment
pre_M = pre_fill %>%
  filter(Experiment == 'M' & sprich > 0) %>%
  select(-c(lm_m:nls_p, lm_pred:nls_p_pred, weighted_lm_exp:weighted_nls_p))

pre_M = pre_M %>% drop_na(bio_filled)

# ratio of NA
sum(is.na(pre_M$biomass)) / nrow(pre_M)
```

```
## [1] 0.1198324
```

The biomass from weighted predicted biomass is 11.98% of total biomass dataset, which is not a large proportion.

5. Why some species some species showed low predictive accuracy

As we mentioned in the Method section, the biomass data was collected in a random strip every year, while the cover data was collected in a fixed area. When a species in a given plot was generally present (i.e., across all years) within the cover area, and generally scarce or absent in the biomass strip, or vice versa, the power for cover to predict biomass will decrease. We already used the average value of cover and biomass data across year for individual plots to fit the models. The best way to build the prediction would be that the cover and the biomass are from the same patch. But unfortunately, we don't have such data. We followed the methods from the previous studies using the data of BioCON experiment to predict the missing biomass data (e.g., Reich et al. 2012, Science; Isbell et al. 2013, PNAS; Mohanbabu et al. 2024, GCB).
