# Supplementary material for: Nitrogen and CO2 enrichment interact to decrease biodiversity impact on complementarity and selection effects
Source: Nat Commun. 2025 Aug 12;16:7445. doi: 10.1038/s41467-025-62691-0 (PMC12343814; doi:10.1038/s41467-025-62691-0)
Supplement: Supplementary file 2 — Reporting Summary [file 41467_2025_62691_MOESM2_ESM.pdf]

nature portfolio

Corresponding author(s): Mengjiao Huang

Last updated by author(s): May 28, 2025

Reporting Summary

Nature Portfolio wishes to improve the reproducibility of the work that we publish. This form provides structure for consistency and transparency in reporting. For further information on Nature Portfolio policies, see our Editorial Policies and the Editorial Policy Checklist.

Statistics

For all statistical analyses, confirm that the following items are present in the figure legend, table legend, main text, or Methods section.

n/a	Confirmed	
☐	☒	The exact sample size (*n*) for each experimental group/condition, given as a discrete number and unit of measurement
☐	☒	A statement on whether measurements were taken from distinct samples or whether the same sample was measured repeatedly
☐	☒	The statistical test(s) used AND whether they are one- or two-sided<br>*Only common tests should be described solely by name; describe more complex techniques in the Methods section.*
☐	☒	A description of all covariates tested
☐	☒	A description of any assumptions or corrections, such as tests of normality and adjustment for multiple comparisons
☐	☒	A full description of the statistical parameters including central tendency (e.g. means) or other basic estimates (e.g. regression coefficient) AND variation (e.g. standard deviation) or associated estimates of uncertainty (e.g. confidence intervals)
☐	☒	For null hypothesis testing, the test statistic (e.g. *F*, *t*, *r*) with confidence intervals, effect sizes, degrees of freedom and *P* value noted<br>*Give P values as exact values whenever suitable.*
☒	☐	For Bayesian analysis, information on the choice of priors and Markov chain Monte Carlo settings
☐	☒	For hierarchical and complex designs, identification of the appropriate level for tests and full reporting of outcomes
☒	☐	Estimates of effect sizes (e.g. Cohen's *d*, Pearson's *r*), indicating how they were calculated

Our web collection on statistics for biologists contains articles on many of the points above.

Software and code

Policy information about availability of computer code

Data collection	No software was used for data collection.
Data analysis	All data analyses were conducted in R version 4.4.0 (www.R-project.org), including 'car', 'nlme', 'piecewiseSEM' packages. R code is available online at Figshare (https://doi.org/10.6084/m9.figshare.26841637.v3).

For manuscripts utilizing custom algorithms or software that are central to the research but not yet described in published literature, software must be made available to editors and reviewers. We strongly encourage code deposition in a community repository (e.g. GitHub). See the Nature Portfolio guidelines for submitting code & software for further information.

Data

Policy information about availability of data

All manuscripts must include a data availability statement. This statement should provide the following information, where applicable:

- Accession codes, unique identifiers, or web links for publicly available datasets
- A description of any restrictions on data availability
- For clinical datasets or third party data, please ensure that the statement adheres to our policy

Source data are provided with this paper at the figshare repository: https://doi.org/10.6084/m9.figshare.26841637.v3. The original plant aboveground biomass data and species percent cover data used in the data synthesis are available from data repositories: https://cedarcreek.umn.edu/research/data. The plant aboveground biomass data used in this study is available at https://portal.edirepository.org/nis/mapbrowse?packageid=knb-lter-cdr.302.newest. The plant species percent cover data is available at https://portal.edirepository.org/nis/mapbrowse?packageid=knb-lter-cdr.301.newest.

Research involving human participants, their data, or biological material

Policy information about studies with human participants or human data. See also policy information about sex, gender (identity/presentation), and sexual orientation and race, ethnicity and racism.

Reporting on sex and gender	N/A
Reporting on race, ethnicity, or other socially relevant groupings	N/A
Population characteristics	N/A
Recruitment	N/A
Ethics oversight	N/A

Note that full information on the approval of the study protocol must also be provided in the manuscript.

Field-specific reporting

Please select the one below that is the best fit for your research. If you are not sure, read the appropriate sections before making your selection.

☐ Life sciences ☐ Behavioural & social sciences ☒ Ecological, evolutionary & environmental sciences

For a reference copy of the document with all sections, see nature.com/documents/nr-reporting-summary-flat.pdf

Ecological, evolutionary & environmental sciences study design

All studies must disclose on these points even when the disclosure is negative.

Study description	This study was based on the BioCON experiment planted in 1997 manipulating biodiversity, CO2 and nitrogen (N) with well-replicated split-plot design. The main random assemblage experiment contained 296 2 m × 2 m plots arranged in six circular 20-m diameter rings. Three rings were exposed to elevated CO2 using free-air CO2 enrichment and three to ambient CO2. Within each ring, half of the plots received N. Plots at each of the four contrasting CO2 and N levels were assigned to four levels of plant species diversity (1, 4, 9, and 16 species) chosen from a pool of 16 perennial grassland species. The experimental design has been published extensively (see Reich et al. 2001, Nature).
Research sample	Aboveground biomass and cover of each species were collected from 1998 and 2000 respectively. There are a lot of publications based on this long-term experiment (see https://scholar.google.com/citations?hl=en&user=DqOiwcQAAAAJ&view_op=list_works).
Sampling strategy	From 1998 to 2021, aboveground biomass was collected by clipping a 10 cm × 100 cm strip in each plot. The cover of each species was estimated in a fixed 50 cm × 100 cm quadrat in each plot.
Data collection	The data was collected by various researchers and students over the 24 years. Plants were cut just above the soil surface. The oven was used to dry plants. The scale was used to weigh the dry plants and Excel was used to record the data.
Timing and spatial scale	Data collection started in 1998 and for this study ended in 2021. Plant biomass was sampled twice per year in June and August from 1998 to 2011, and once per year in August since 2012. The cover of each species was estimated twice per year in June and August from 2000 to 2021.
Data exclusions	This study used the data in August from 1998 to 2021 of the 296 plots in the main experiment. A total of 48 out of 296 plots were used for additional experiments, with 24 plots for precipitation treatment from 2007 and 24 plots for precipitation ambient. Within each of the two precipitation levels, half of the plots received warming treatment from 2012 (see Reich et al. 2020, Nature Geoscience). Therefore, we excluded 24 plots with a precipitation reduction treatment from 2007 and another 12 plots with warming treatment from 2012. When calculating complementarity effects and selection effects, We excluded species with a monoculture

	biomass less than 2.5 g m-2 in a given plot and a given year because relative yield can approach infinity with small monoculture biomass values (see details in Methods section).
Reproducibility	The experiment is repeatable over the 24 years. The source data and the R code is available at Figshare (https://doi.org/10.6084/m9.figshare.26841637.v3).
Randomization	The main BioCON experiment is a split-plot arrangement of treatments in a completely randomized design. CO2 treatment is the whole-plot factor and is replicated three times among the six rings. The subplot factors of species number and N treatment were assigned randomly and replicated in individual plots among the six rings.
Blinding	NA

Did the study involve field work? ☒ Yes ☐ No

Field work, collection and transport

Field conditions	The region has a continental climate with warmer summer (average temperature of 22 °C in July) and cold winter (average temperature of -11 °C in January), and average annual precipitation is 660 mm.
Location	The BioCON experiment is at the Cedar Creek Ecosystem Science Reserve, Minnesota, United States (45°40′N, 93°18′W).
Access & import/export	Access to the site and data collection followed standard practices and complied the laws.
Disturbance	This study did not cause any environmental disturbance.

Reporting for specific materials, systems and methods

We require information from authors about some types of materials, experimental systems and methods used in many studies. Here, indicate whether each material, system or method listed is relevant to your study. If you are not sure if a list item applies to your research, read the appropriate section before selecting a response.

Materials & experimental systems

n/a	Involved in the study	
☒	☐	Antibodies
☒	☐	Eukaryotic cell lines
☒	☐	Palaeontology and archaeology
☒	☐	Animals and other organisms
☒	☐	Clinical data
☒	☐	Dual use research of concern
☐	☒	Plants

Methods

n/a	Involved in the study	
☒	☐	ChIP-seq
☒	☐	Flow cytometry
☒	☐	MRI-based neuroimaging

Dual use research of concern

Policy information about dual use research of concern

Hazards

Could the accidental, deliberate or reckless misuse of agents or technologies generated in the work, or the application of information presented in the manuscript, pose a threat to:

No	Yes	
x		Public health
x		National security
x		Crops and/or livestock
x		Ecosystems
x		Any other significant area

Experiments of concern

Does the work involve any of these experiments of concern:

No	Yes	
x		Demonstrate how to render a vaccine ineffective
x		Confer resistance to therapeutically useful antibiotics or antiviral agents
x		Enhance the virulence of a pathogen or render a nonpathogen virulent
x		Increase transmissibility of a pathogen
x		Alter the host range of a pathogen
x		Enable evasion of diagnostic/detection modalities
x		Enable the weaponization of a biological agent or toxin
x		Any other potentially harmful combination of experiments and agents

Plants

Seed stocks	Plants were cut just above the soil surface, dried and weighed to estimate the aboveground biomass.
Novel plant genotypes	NA
Authentication	NA
